# Behavioral Lifestyle Interventions for Weight Loss in Overweight or Obese Patients with Type 2 Diabetes: A Systematic Review of the Literature

**DOI:** 10.1007/s13679-024-00552-5

**Published:** 2024-03-04

**Authors:** Sara Gostoli, Giulia Raimondi, Alexandra Paula Popa, Micaela Giovannini, Giada Benasi, Chiara Rafanelli

**Affiliations:** 1 Department of Psychology “Renzo Canestrari”, University of Bologna, Viale Berti Pichat 5, 40127 Bologna, Italy; 2 Department of Medicine, Division of General Medicine, Columbia University Irving Medical Center, 622 West 168th Street, 10032 New York, NY United States

**Keywords:** Behavioral intervention, Weight loss, Type 2 diabetes, Lifestyle, Overweight, Obesity

## Abstract

**Purpose of Review:**

Around 80–90% of patients with type 2 diabetes mellitus (T2DM) are overweight or obese, presenting a greater risk for serious health complications and mortality. Thus, weight loss represents a main goal for T2DM management. Although behavioral lifestyle interventions (BLIs) could help promoting weight loss in T2DM patients with overweight or obesity, their effectiveness is still controversial. This systematic review offers an updated and comprehensive picture of BLIs according to Michie’s classification in T2DM patients with overweight or obesity and identifies possible factors (related to both patients and interventions) associated with weight loss. The PRISMA guidelines were followed. The literature search till March 2023 indicated 31 studies involving 42 different BLIs.

**Recent Findings:**

Our findings suggest that structured BLIs, characterized by frequent feedback and support, can lead to a clinically meaningful 5% weight loss, regardless of specific behavioral, diet, and physical activity components.

**Summary:**

Further research should address methodological issues and heterogeneity of interventions, also considering the effect of pharmacological therapies on weight reduction. Lastly, more attention should be paid to the long-term effectiveness of behavioral lifestyle interventions and to the relationship between weight loss and diabetes.

**Supplementary Information:**

The online version contains supplementary material available at 10.1007/s13679-024-00552-5.

## Introduction

Overweight and obesity affect 38% of the global population [1]. These conditions are steadily and continuously rising even in low-income nations, whereas an increasing number of high- and middle-income countries are currently experiencing an epidemic of severe obesity, with expected doubled prevalence (from 10 to 20%) between 2020 and 2035 [2]. Overweight and obesity are associated with metabolic syndrome [2] and represent major risk factors for the development of type 2 diabetes mellitus (T2DM) [1–3]. The prevalence of T2DM increases linearly with body mass index (BMI) [4–7], and around 80–90% of diabetic patients are overweight or obese [8], presenting a greater risk for serious health complications and mortality [2, 8, 9].

Given the significant increase in the prevalence of both T2DM and obesity, it is of particular importance to provide patients with diabetes with effective weight loss interventions [3]. Indeed, weight loss is considered a main goal for diabetes management [3, 10–12], and even a modest weight loss of 5–10% of the initial weight can improve health outcomes, such as levels of glycemia, lipids, and blood pressure [13, 14].

Lifestyle modification represents a key strategy for promoting weight loss and an essential part of most interventions. Behavioral lifestyle interventions often involve a combination of dietary, physical activity, and behavioral components, which involve strategies to promote changes in diet and physical activity [7, 15–17]. Although these interventions have a positive impact on physical health and quality of life [9, 15, 18, 19], evidence about their efficacy in terms of weight loss is still controversial [10, 19–24] and results are difficult to maintain in the long term [25, 26].

Several systematic reviews and meta-analyses have examined the effectiveness of lifestyle-based interventions in promoting weight loss in overweight or obese patients with T2DM. However, some of these systematic reviews and meta-analyses are outdated [19–21]: in some of them, behavioral strategies were not the essential component of the lifestyle intervention [10, 19, 21, 22]; some used restricted study selection criteria (e.g., study duration ≥ 1 year) [10, 19, 22]; in most of them, overweight or obesity were not inclusion criteria [19, 22–24]. Finally, to the best of our knowledge, no systematic review focusing on behavioral lifestyle interventions have used the taxonomy proposed by Michie and colleagues [27], which was created in order to obtain a detailed and consensually agreed structure classification of techniques used in behavior change interventions. Specifically, this taxonomy was built by means of an empirical approach based on content analysis, independently from any theoretical framework. This approach benefits both clinical interventions, by facilitating the implementation of those techniques found to be the most effective, and systematic reviews, since it allows the correct identification and synthesis of distinct methods aimed at behavior change, avoiding overlaps and/or use of redundant terminology.

Therefore, the first aim of this systematic review is to provide an updated and comprehensive picture of the characteristics and effectiveness of behavioral lifestyle interventions in overweight or obese adult patients with T2DM, using Michie and collaborators’ taxonomy [27]. The second aim is to identify all potential factors (i.e., related to the patients and/or interventions) that can either positively or negatively affect the efficacy of behavioral lifestyle interventions in promoting weight loss in overweight or obese adult patients with T2DM. The primary outcome was weight loss, at least at post-treatment and/or at follow-up.

## Methods

### Search Strategy

PRISMA guidelines [28] were followed. A comprehensive search of the literature from 2013, after the publication of Michie and collaborators’ classification [27] of behavioral lifestyle interventions, up to December 2023 was performed in PubMed, PsycInfo, and Scopus Library. The following search terms have been used: “obesity” OR “obese” OR “overweight” AND “diabetes mellitus” OR “type 2 diabetes” OR “diabetes” AND “behavioural lifestyle intervention” OR “behavioral lifestyle intervention” OR “behavioural intervention” OR “behavioral intervention” OR “psychological intervention” OR “lifestyle intervention” OR “Behavior Change Techniques” OR “BCT” OR “behaviour change intervention” OR “behavior change intervention” OR “weight loss intervention” OR “weight loss”. Reference lists from relevant studies and reviews were analyzed for additional clinical trials (Table S1).

### Study Selection

A first screening of title and abstract was performed, and the full text of potentially eligible studies was analyzed. Search, selection, and analysis of the selected studies were performed independently by two reviewers; disagreements were resolved by consensus among these primary raters and a senior investigator.

Only articles published in English were considered for inclusion. Specifically, articles were eligible if they included at least one behavioral lifestyle intervention for weight loss involving at least one behavioral component, in a sample of adult (18 + years old) patients with T2DM and overweight or obesity (BMI ≥ 25).

We included studies of various designs that involved weight change (e.g., in kg or BMI, or as percentage of weight loss or gain from baseline) within outcome measures. Studies were excluded if (a) participants were < 18 years old, (b) had a BMI < 25, (c) were affected by mental disorders or (d) by other forms of diabetes (e.g., pre-diabetes, gestational diabetes, type 1 diabetes), and if (e) no clear data about sample characteristics, such as age and BMI, were available. We also excluded studies (f) not reporting quantitative results from inferential analyses about weight change within and/or between intervention groups, (g) in which behavioral intervention was only administered in combination with another treatment (e.g., bariatric surgery and weight loss medication), and (h) focusing on diabetes prevention or post-bariatric surgery.

Behavioral interventions were analyzed using Michie et al. [27] taxonomy, which is hierarchically organized and includes 93 distinct behavior change techniques (BCTs), clustered into 16 groups. BCT indicates an observable, replicable, and irreducible component of an intervention designed to alter or redirect causal processes that regulate behavior. The classification process that allows to extract information about intervention content, replicate interventions, and evaluate existing ones has been described [27].

### Data Extraction

Data were independently extracted by both reviewers by means of a precoded form. They included study design, setting and duration, demographics and sample characteristics (i.e., age, gender distribution, BMI, primary diagnosis and comorbidities, sample size), type and time of assessment, comparison groups, duration, intervention components analyzed using Michie’s taxonomy [27], weight, anthropometric, metabolic and psychological outcomes.

### Data Synthesis

Considering the highly heterogeneous interventions, controls, and outcomes, a meta-analysis was not deemed to be appropriate.

## Results

**Fig. 1:**
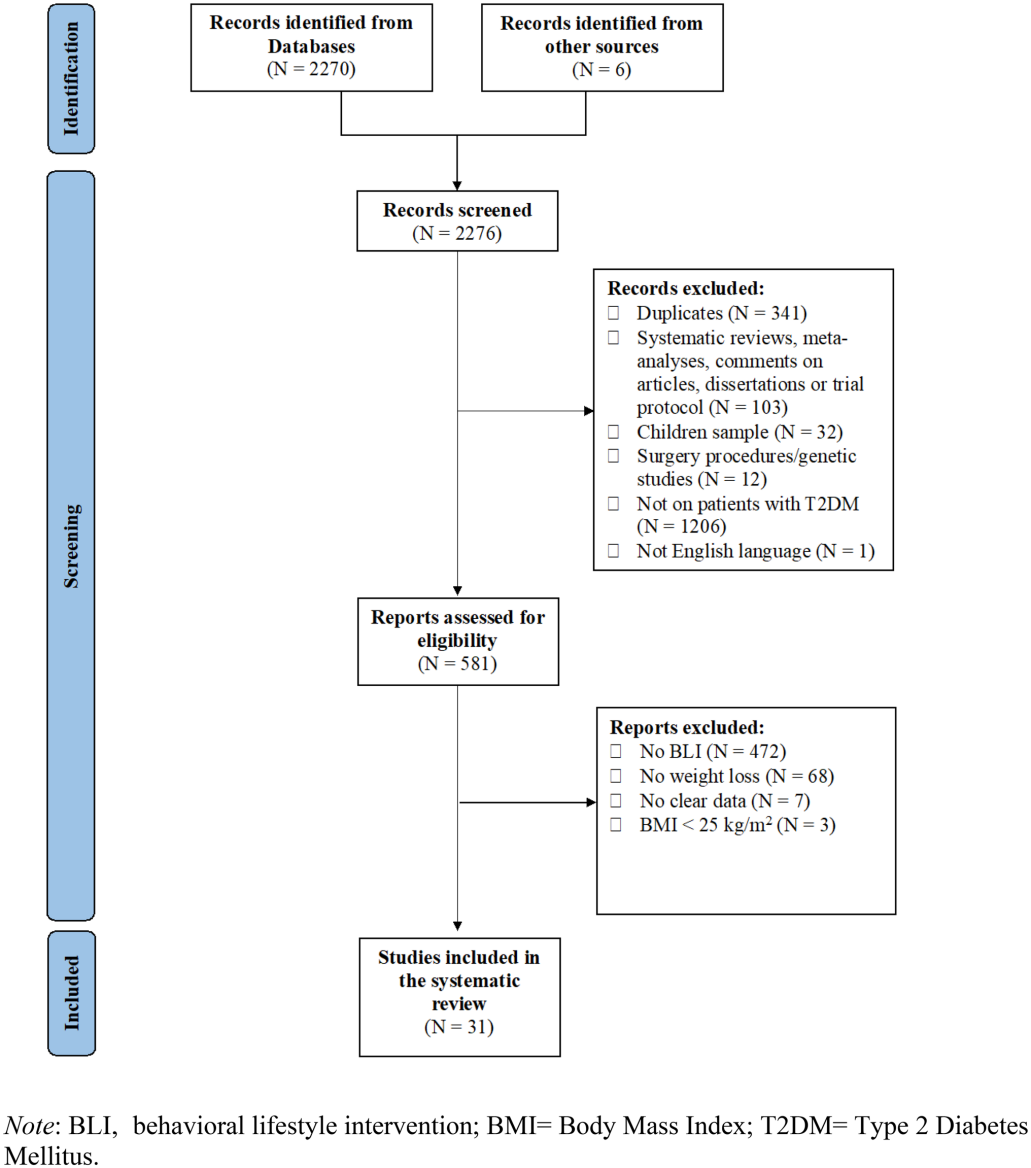
PRISMA flow chart of study selection

**Fig. 2:**
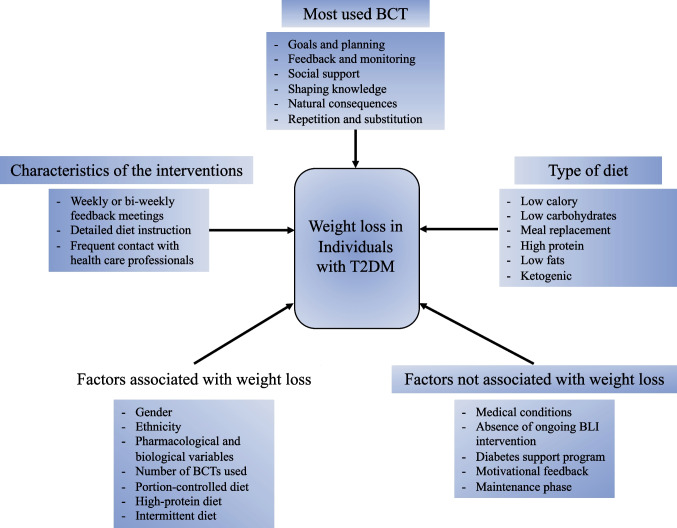
Synopsis of main findings of the present systematic review

At the end of the screening process, 31 studies, which reported a total of 42 behavioral lifestyle interventions, were included in the review (see Fig. 1 for a detailed description of the study selection process). The main findings of the present systematic review are summarized in Fig. 2.

### Characteristics of the Studies

The included studies were published between 2013 and 2023 (Tables S2 and S3). All studies were randomized controlled trials (RCTs). Among the RCTs identified, three were feasibility trials [29, 30, 31•] and three pilot studies [32–34]. Most studies adopted a two-group design, except for 4 studies, which adopted a three-group design [29, 30, 33, 34].

Four additional studies compared “device-based” behavioral lifestyle interventions to educational interventions [29, 30, 35•, 36]. Ten studies [37–43, 44••, 45, 46] belonged to the Look AHEAD trial. Besides the Look AHEAD trial, seven additional studies compared a behavioral lifestyle intervention vs. usual care control group [31•, 33, 34, 47–49, 50••, 51, ] and a no-treatment control group [52]. Five studies compared interventions consisting of different behavioral techniques associated with specific diets [32, 53–57, 58••], and 2 studies compared behavioral lifestyle intervention vs usual care in subgroups of ethnic minorities [36, 38].

Studies included a follow-up evaluation ranging from 1 month to almost 10 years after the end of the intervention. Excluding the Look AHEAD studies [38–43, 44••, 45, 46], which included the longest follow-ups, other research involved follow-up of 1 month [36], 6 months [29, 31•, 35•, 49], 12 months [31•, 47, 48], and 48 months [48].

### Characteristics of the Sample

The 31 studies included a total of 7272 participants, of whom 5145 were part of the Look AHEAD study. The sample size ranged from 26 to 5145. The mean age of the sample ranged from 36.5 to 69 years at baseline (Tables S2 and S3). Excluding a single study in which the sample was exclusively represented by women [36], the percentage of women in the remaining studies ranged from 34 to 75%. The mean BMI ranged from 31.6 to 38.1.

In 8 studies [35•, 39, 40, 42, 43, 45, 48, 50••], the sample reported having comorbidities with other medical conditions, such as chronic kidney disease [35•], obstructive sleep apnea [48], and cardiovascular disease [39, 40, 42, 43, 45, 50••]. More specifically, the sample in the study of Delahanty et al. [50••] reported the presence of other medical conditions, such as hypertension, hyperlipidemia, retinopathy, neuropathy, proteinuria, coronary artery disease, and congestive heart failure.

In 6 studies [40, 42, 47, 54, 56, 57], participants reported using pharmacological management, specifically they reported taking metformin [54], sulphonylureas [54], GLP-1 agonists [54], DPP-4 inhibitors [54], lipid lowering medication [54], antihypertensive medication [54], ACE inhibitor [56], angiotensin receptor blocker [56], diuretic [56], calcium-channel blocker [56], statin [56], antiplatelet drug [56], oral antidiabetic [57], antihyperglycemic medicines [47], oral hypoglycemic medication [40], and cholesterol medication [42].

### Behavioral Lifestyle Intervention Design

Overall, 42 behavioral lifestyle interventions were identified among the 31 studies (Tables S1 and S3).

Even if all studies included behavioral components in association with diet and/or physical activity, the interventions were heterogeneous in the duration, frequency of contacts, staff, techniques, and type of intervention. Interventions’ duration ranged from 9 weeks to 5 years, with daily, weekly, bi-weekly, and/or monthly contacts. In most interventions, the frequency of contacts tended to be more intense at the beginning of the program and then diminished over time.

Additionally, eleven studies [30, 38–43, 44••, 45, 53, 54] included the evaluation of a maintenance phase, in which the longest phase lasted 12 weeks after the end of an intensive phase. The maintenance phase was meant to assess the preservation and consolidation of the achieved results.

Most interventions were performed by dieticians and exercise trainers. Other interventionists included physicians, nurses, physical therapists, physiologists, clinical psychologists, social workers, diabetes educators, and trained research staff. Sessions were generally delivered in-person and could be individual sessions, group sessions, or a combination of both.

There were 10 studies belonging to a major clinical trial, the Look AHEAD trial [37–43, 44••, 45, 46] which is a multicentric RCT on adults with T2DM and overweight/obesity (BMI ≥ 25, or BMI ≥ 27 if taking insulin therapy), which compared an intensive lifestyle intervention (ILI) to achieve weight loss through caloric restriction and physical activity, with a Diabetes Support and Education (DSE) group. The study began in 2001 and included over 5000 participants (2570 in the ILI group and 2575 in the DSE group). The interventions phase lasted 4 years. Outcomes were assessed once/twice a year, from baseline up to 9.6 years (median) of follow-up.

### Behavioral Component

A variety of different behavioral techniques, proposed by Michie’s taxonomy [27], were used in the behavioral lifestyle interventions included in the current work, and the most used taxonomy groups of interventions were *goals and planning*, *feedback and monitoring*, *social support*, *shaping knowledge*, *natural consequences*, and *repetition and substitution.* Specifically, the *goals and planning* taxonomy group was the most frequent behavioral protocols in the studies and mainly provided a description of objectives in terms of the behaviors to be implemented, the results to be achieved, the review of these objectives, problem solving, and a detailed description of the performance of the behavior. In the taxonomy group *feedback and monitoring*, the characterizing techniques were monitoring and/or self-monitoring of behaviors and reached goals, and biofeedback. *Social support* mainly comprised general support and, to a lesser extent, emotional support. With respect to *shaping knowledge* group, all the participants in the studies were given instructions on how to perform the behavior relating to diet, physical activity, and lifestyle. Considering *natural consequences* category, ingredients focused on education about health consequences of desired behaviors, also using specific methods to highlight the consequences of the performance of behavior in order to make it more memorable and, to a lesser extent, is given space to the identification and monitoring of emotional consequences. Finally, considering the group *repetition and substitution*, in all the considered studies the repetitive practice of one behavior was solicited in order to form new habits and replace old less or non-functional ones (Table S1).

Counseling meetings were intended to increase motivation and offer general support in making lifestyle changes, both during the intervention and follow-ups, when this was the case [30, 34, 48]. Educational meetings included diet, weight, and diabetes self-management education. Most interventions incorporated cognitive-behavioral therapy strategies for diabetes management and weight loss, including self-monitoring and/or monitoring by others (e.g., dietician) of weight, dietary and calorie intake, blood glucose, and adherence; goal setting; problem solving; and homework assignments.

### Diet Component

General diet recommendations were provided in 23 studies [29, 30, 33, 34, 35•, 36–43, 44••, 45, 47–49, 50••, 51, 52, 59], while more specific diet instructions were provided in 7 studies [32, 46, 53–57]. These latter included calorie restriction and/or management of macronutrients intake, such as carbohydrates, proteins, fibers, and fats (saturated and unsaturated). Furthermore, 7 studies used meal replacement [30, 34, 41, 43, 45, 48, 50••].

Specifically, Carter et al. [53] compared a 500–600 kcal/day diet for 2 days/week and the usual diet for 5 days/week to a continuous 1200–1500 kcal/day diet. Watson et al. [54] compared a higher protein diet with a higher carbohydrate diet. Both diets were followed by a 12-week eucaloric maintenance phase. Otten et al. [55, 56] and Stomby et al. [58••] tested the efficacy of a Paleolithic diet alone (PD) or associated with a supervised exercise intervention (PDEX). The Paleolithic diet is a type of diet that excludes the intake of diary food, salt, refined sugar, and grains and simultaneously increases the intake of vegetables, lean meat fish, and nuts [60]. Ziegler et al. [32] compared the efficacy of a diet high in cereal fiber, free of red meat, and high in coffee versus a diet low in fiber, high in red meat, and coffee. Goday et al. [57] compared a ketogenic diet and a very low-calorie diet (VLCK) with a standard low-calorie diet.

### Physical Activity Component

A physical activity component was present in all 31 behavioral lifestyle interventions (Tables S2 and S3): 11 studies encouraged physical activity without any specific instruction [29, 30, 32–34, 36, 50••, 51, 53, 57, 59], while 20 studies provided clear indications on the type, intensity, duration, and/or frequency of physical activity [35•, 37, 39–43, 44••, 45–49, 52, 54–56, 58••, 59]. The intensity of physical activity varied from low intensity walk [38, 43, 45, 48, 49, 51, 58••] to moderately intense activity [35•, 39–42, 44••, 46, 47, 52, 54–56], and it was gradually increased in most cases. Duration, number of calories to consume, effort level (e.g., pulse control), and type of activity (e.g., walking, running, swimming, resistance exercises, aerobics) were specified.

Physical activity sessions could be carried out in an individual, group, or mixed individual/group setting. In the case of group sessions, activities were supervised by experienced personal trainers or physiotherapists [37, 55, 56, 58••].

### Device-Based Intervention Versus In-Person Intervention

The behavioral lifestyle interventions provided by the studies included in the current work are very different within each other. The main difference among the studies is the way the behavioral lifestyle intervention was conducted. For example, 4 studies [29, 30, 35•, 36] delivered a “device-based” lifestyle interventions, which consisted of lifestyle intervention delivered through phone calls [36], videocalls [35•], or by adopting a small wearable device [30] that recorded physical activity and nutritional intake, providing graphical feedback of these measures. The remaining studies [31•, 32–34, 37–43, 44••, 45–49, 52–57, 58••] delivered “in person” lifestyle interventions, meaning that patients had to participate in person to group sessions and to individual assessment sessions. Two study [29, 50••] used a combination of both “device-based” and “in person” lifestyle interventions. In general, among the studies involving the “in person” lifestyle intervention, 22 focused not only on diet and physical activity but also on goals setting, problem solving, self-monitoring, cognitive restructuring, and relapses prevention, while the remaining 8 studies [32, 52–57, 58••] exclusively focused on the efficacy of different types of diets and exercise (Table S1).

Specifically, Wang et al. [29] compared the efficacy of a device-based behavioral lifestyle intervention vs a paper-based self-monitoring intervention of multiple behaviors on weight loss. Bentley et al. [30] used a device which monitored physical activity and calorie intake. Participants had to also send weekly emails to the research team describing how the week went (i.e., “event diaries”) and received tailored response that aimed at boosting the participant’s motivation. Finally, in St-Jules et al. [35•] and Lutes et al. [36], the experimental group received monthly emails about recommendations on how to achieve the interventions’ targets.

### Structured Versus Less Structured Interventions

Twenty-four studies included a more structured behavioral intervention [29, 30, 31•, 32–34, 35•, 36–43, 44••, 45, 46, 49, 50••, 51, 53, 57], meaning that those studies gave a specific program regarding the targets of the interventions, diet, calorie intake, exercise program, session attendance, and self-monitoring of behavior change, while the other 7 studies included a less structured behavioral intervention [47, 48, 52, 54–56], where more general and not specific recommendations were given. In all the studies, the behavioral interventions were delivered by different health care practitioners (i.e., physicians, psychologists, dieticians).

### Individual Versus Group Interventions

Two studies [31•, 36] conducted the behavioral lifestyle intervention through individual sessions, 3 studies [29, 49, 51] used group sessions, while 16 studies [30, 33, 34, 35•, 37–43, 44••, 45–48, 50••, 55–57, 59] used both individual and group sessions. In the latter case, the interventions were delivered differently among the groups included [34, 37–43, 44••, 45, 46, 48, 50••, 51, 59], meaning that one group underwent group sessions, while the other individual sessions. In the individual sessions were also included interventions delivered through phone calls and video calls [31•, 35•, 36, 59] regarding the device-based trials. In 11 studies [30, 32, 35•, 37–43, 44••, 45, 46], individual meetings were also used to obtain anthropometric measurement (e.g., weight change, blood pressure) and to assess whether there were any difficulties of feedback related to the dietary program. Finally, 5 studies [52–56] did not specify whether the intervention (e.g., dietary counseling, exercise training) was conducted individually or in group.

Specifically, in Lutes et al. [36], the intervention lasted 12 months, and participants of the experimental group underwent 16 phone-based individual sessions, while the control group received 16 educational mailings. In Wang et al. [29] and Foster et al. [49], both groups took part in a 6-month intervention; specifically, in Wang et al. [29], both groups attended 11 group sessions, while in Foster et al. [49], both groups attended 9 group sessions. In Gamiochipi et al. [51], participants of both groups received 16 group sessions on behavior modification over the course of 6 months. Finally, in Stomby et al. [58••], both groups received five group sessions during the course of the 12-week intervention.

In Kuna et al. [48], participants in the experimental group attended 3 group sessions during the first 2 years and then, starting from year 2 until year 4, the intervention was delivered monthly and individually, whereas the control group attended 3 sessions annually. In Delahanty et al. [33, 34, 50••], participants in the control group received 1-hour long individual sessions followed by 20–40 min follow-up sessions arranged according to the dietician’s discretion, while the experimental group attended 19 weekly 1.5-h long group sessions, during the 6 months intervention. Differently, in Delahanty et al. [50••], participants in the experimental group received 25 1.5-h long-group sessions, of which 14 weekly sessions, 5 bi-weekly sessions, 6 monthly sessions, and up to 3 optional individual sessions, over the course of 1 year. Finally, in the Look AHEAD study [37–43, 44••, 45, 46], the interventions phase lasted 4 years, with weekly meetings: the experimental group underwent 3 group meetings and 1 individual counseling per month during the first 2 years, while the control group was administered only monthly group sessions.

### Effects on Weight Loss

In general, among all the included studies, 25 reported a weight loss after the behavioral lifestyle intervention. Among the studies that compared different dietary approaches, positive results were found in six studies [32, 54, 57, 58••]. Similar results were found by Watson et al. [54], Ziegler et al. [32], and Stomby et al. [58••]. Specifically, Watson et al. [54] showed that both higher protein and higher carbohydrate diets were effective on weight loss after 24 weeks, with 8 kg (− 8.22% of initial weight) lost in the group with the highest protein intake and − 7.6 kg (7.49% of initial weight) lost in the group with the highest carbohydrate intake. Ziegler et al. [32] found that a diet high in cereal fiber, free of red meat, and high in coffee was just as effective on weight loss as a diet low in fiber, high in red meat, and coffee-free after 8 weeks. Stomby et al. [58••] found that both a Paleolithic diet alone (PD) or associated with a supervised exercise intervention (PDEX) result in a clinically significant weight loss, in terms of BMI (PD: − 8%; PDEX: − 7%; *p* < 0.05) at 12 weeks of the trial. Similar results were found in Otten et al. [55, 56] study in which there was a clinically significant (*p* < 0.001) weight loss for both the PD group (− 7.1 kg) and PDEX group (− 7 kg) at 12 weeks of the trial. Finally, Goday et al. [57] reported that a very low-calorie diet (VLCK) was associated with a greater weight loss (*p* < 0.001) compared to a standard low-calorie diet. Finally, in the Look AHEAD study [38–43, 44••, 45, 46, 51, 59], 50% of the participants in the ILI group lost at least 5% of their initial weight, and 26.9% lost at least 10%, after 8 years. Considering the DSE group, 35.7% of participants lost at least 5% of initial weight, and 17.2% lost at least 10% [40]. At the end of the study, the ILI group lost an average of 6% of the initial weight, while the DSE group 3.5% [46].

Lutes et al. [36] found that individuals who underwent a phone-based lifestyle intervention had a clinically and significant weight loss (− 1.35 ± 6.22 vs. + 0.07 ± 2.01 kg; *p* < 0.001). In the studies by Moncrieft et al. [47], Foster et al. [49], Delahanty et al. [33, 34, 50••], and Gamiochipi et al. [51], participants of the experimental group lost a clinically (≥ 5%) and statistically significant (all *p* < 0.05) amount of the initial weight after treatment compared to controls. In Moncrieft et al. [47], the intervention effect on weight was statistically significant (*b* =  − 0.322, *p* = 0.010), with an average of 0.32 kg of weight loss per month over the first 6 months of intervention, which was maintained through the 12-month assessment. In Kuna et al. [48], the intervention group had a significantly greater weight loss than the diabetes support and education group at 1, 2, and 4 years. The difference in body weight between the two groups was − 10.8 kg, − 6.7 kg, and − 4.4 kg at 1, 2, and 4 years, respectively. In Hanakonen et al. [59], participants who self-reported using all BCTs lost more weight, in terms of BMI (− 1.18 kg/m^2^), compared to those who used 10 or fewer BCTs (− 0.10 kg/m^2^; *p* = 0.013). For example, participants who applied the behavioral component of goals setting to both eating a low-fat diet and increasing physical activity lost significantly more weight, in terms of BMI (− 0.88 kg/m^2^), than participants who used goals setting only for dietary purpose (− 0.08 kg/m^2^) or physical activity (− 0.23 kg/m^2^), separately. In Foster et al. [49], participants who were assigned to a lifestyle intervention, which included a portion-controlled diet, lost 7.3 kg compared to 2.2 kg lost by the control group who followed a diabetes self-management education. In Delahanty et al. [33, 34, 50••], mean weight loss was 6.6 kg in the experimental group and 2.1 kg in the control group (*p* < 0.001). The weight loss of the experimental group remained significantly greater after 1 year. In Gamiochipi et al. [51], weight loss was 2.18 kg in the experimental group and 0.85 kg in the control group (*p* = 0.04). Osama et al. [52] reported that the combination of aerobic exercise training and a dietary program in 3 months led to a statistically significant (*p* < 0.05) weight loss in terms of BMI (30.13 kg/m^2^) compared to controls (33.45 kg/m^2^). Finally, although Benasi et al. [31•] found no statistically significant weight loss change between both the experimental groups (i.e., Well-Being Therapy + Lifestyle Intervention and Lifestyle Intervention), a statistically significant difference was found within each group after the intervention and at 6-month follow-up (WBT + Lifestyle: T1 =  − 2.5 kg, T2 =  − 2.2 kg; Lifestyle intervention: T1 =  − 1.7 kg, T2 =  − 1.6 kg).

The remaining 4 studies [29, 30, 35•, 53] found no difference in weight loss in the experimental group compared to the control group after treatment. Wang et al. [29] found that even though at 6 months participants in the device-based group had an average weight loss of 1.8%, while the paper group and control group reported a 4% and 1.6% weight gain, respectively, there were no statistically significant differences among the three groups on weight changes over time (*p* = 0.48). Bentley et al. [30] reported a weight loss at week 16 of the trial for the groups undergone the device-based intervention (intervention groups of − 3.3 kg and − 3.0 kg, compared to controls + 0.7 kg). However, since the sample sizes were too small, Bentley et al. [30] did not conduct any inferential analyses (statistical *p* are not reported); these results should be thus interpreted with caution. Carter et al. [53] found that the use of a low-calorie diet for 2 days/week and the usual diet for 5 days/week in the experimental group compared to a continuous energy restriction diet in the control group did not result in weight loss. Instead, participants regained 33% of their weight lost, between 12 and 24 months after treatment. Finally, St-Jules et al. [35•] reported that even though the experimental group had a significant weight loss compared to the control group at 3 months of the trial (*p* = 0.08), it was no longer significant at 6 months (*p* = 0.39).

The mentioned interventions highlighted different factors that seem to be positively or negatively associated with weight loss.

### Factors Associated with Weight Loss

Among the studies that reported a significant weight loss for individual with T2DM undergoing BLI, some factors that might have favored weight loss could be identified.

The factors that were indicated as associated with weight loss were (i) sociodemographic variables [38] (i.e., gender, ethnicity), (ii) pharmaceutical variables [36, 40, 42, 47, 54, 56, 57] (e.g., metformin, insulin), (iii) biological variables [55] (i.e., levels of plasma fetuin-A, which is a protein secreted by the liver and induces insulin resistance [61]), (iv) variables related to the interventions [47, 59] (i.e., number of sessions attended, number and type of behavioral change techniques (BCTs), (v) diet-specific variables [53, 54, 57] (i.e., portion-controlled diet, diet high in proteins, intermittent diet).

West et al. [38] reported that (i) gender in relation to both individual session and group session attendance seems to be another factor affecting weight loss for ethnic minorities. For Hispanic men, individual session attendance was the stronger predictor of weight loss in the first 4 years, while group session attendance was of major importance for Hispanic women. West et al. [38] also highlighted that (i) ethnicity in relation to individual session attendance seems to be a key factor for weight loss for ethnic minorities. In all the studies [36, 40, 42, 47, 54, 56, 57] that reported participants using (ii) pharmacological management, findings for weight loss were reported. Specifically, Lutes et al. [36] found that insulin was negatively associated with weight loss: post-hoc analyses demonstrated that individuals who were not using insulin had a significant greater reduction of weight (− 2.36 ± 6.59 vs. − 1.64 ± 4.36 kg; *p* = 0.003) compared to controls not on insulin. In Otten et al. [55], (iii) a reduction in plasma fetuin-A levels was associated with a decrease in liver fat. Specifically, individual sessions attendance was a strong predictor of weight loss among African-American. Moncrieft et al. [47] highlighted that (iv) at least 7 sessions need to be attended to show greater weight loss. Hankonen et al. [59] found that (iv) the number (i.e., all BCTs) and type (i.e., goal setting, goal review, and social support) of BCTs used were significantly associated with weight loss. According to Foster et al. [49], (v) the presence of a portion-controlled diet (PCD) into the BLI helped individuals reach a clinically meaningful weight loss in fewer session than required if a traditional low-calorie diet was used. The use of (v) a diet high in proteins seems to lead to a greater weight loss compared to a standard care nutritional intervention, as highlighted by Goday et al. [57] and Watson et al. [54]. Finally, according to Carter et al. [53], (v) intermittent diet was associated with weight loss maintenance after treatment (at 24 months of follow-up): the majority of participants maintained weight loss and adjusted well to the reduced energy intake required by the diet. In fact, participants reported to keep using intermittent diet principles in order to assist weight maintenance after the end of the trial.

### Factors Not Associated with Weight Loss

Even though the majority of the included studies reported a significant weight loss after treatment for individuals with T2DM, some studies highlighted the presence of factors that do not seem to be associated with weight loss.

The factors indicated by the studies were (i) other medical condition [35•, 39, 40, 42, 43, 45, 48, 50••] (e.g., cardiovascular disease), (ii) variables related to the interventions [30, 35•, 53] (i.e., the absence of ongoing BLI intervention and diabetes support program over time, motivational feedback, focusing only on the “target” of the intervention, such as weight loss). No pharmacological factors were indicated by the studies as potential factors hampering weight loss.

The presence of (i) other medical conditions, such as obstructive sleep apnea [48] and cardiovascular disease [39, 41, 45, 50••], does not seem to be a factor that hampers weight loss because all studies in which participants reported these medical conditions reported a significant weight loss. However, only in the study of St-Jules et al. [35•], in which patients reported a history of chronic kidney disease, there was not a significant weight loss in both groups. According to Bentley et al. [30], (ii) receiving motivational feedback emails does seem to cause more adherence to treatment, but does not appear to affect the amount of weight loss. According to Carter et al. [53], (ii) a factor that could impede weight loss after BLI is the absence of an ongoing BLI intervention and dietetic support program over time. In other words, the effects of a BLI cannot be maintained if there is no continuity of the intervention over time, and weight regain is frequently observed [53]. Lastly, as highlighted by St-Jules et al. [35•], (ii) education on the intervention target alone is not enough for engaging in lifestyle behavior change. Specifically, the authors reported a non-significant weight loss in both control and experimental groups.

Characteristics and findings of the major studies included in the present systematic review are summarized in Table 1.

**Table 1:** Characteristics and findings of major studies

**Authors, year**	**Sample size (%F)**	**Population**	**Characteristics of the intervention** **(frequency, duration, setting, type of staff)**	**Assessment and follow-up**	**Major findings**	
					**Weight and/or BMI**	**Conclusions**
Benasi et al. 2022 [31•]	58 (40%)	Adults (age range: 18–65 years; mean age = 55.5 (6.6) years), BMI ≥ 25 kg/m^2^, 4 (2–5) with medical comorbidities, 22% taking insulin, with type 2 diabetes G1 (WBT-lifestyle, *n* = 30) G2 (lifestyle alone, *n* = 28)	G1 (WBT-lifestyle): - Frequency: 4 weekly 1 h long individual sessions of WBT; 12 weekly individual sessions of lifestyle intervention, 4 of which were 1 h long and conducted in person, the remaining 8 were 30 min long and delivered through telephone calls - Setting: individual - Staff: psychotherapists specialized in WBT, clinical psychologists G2 (lifestyle alone): - Frequency: 12 weekly individual sessions, 4 of which were 1 h long and conducted in person, the remaining 8 were 30 min long and delivered through telephone calls - Setting: individual - Staff: clinical psychologists	Baseline, 4 months, 6 months, and 12 months	Weight (kg) at baseline: G1: 94.8 (23.4) G2: 95.6 (19.1) Weight (kg) at 4 months: G1: 96.4 (26.1) G2: 93.2 (17.2) *P* = 0.50 Weight (kg) at 6 months: G1: 97.1 (26.7) G2: 93.3 (17.9) *P* = 0.62 Weight (kg) at 12 months: G1: 93.0 (19.9) G2: 90.6 (17.6) *P* = 0.48	At post-treatment and 6-month follow-up, within-group analysis revealed that both type 2 diabetes mellitus patients who underwent a sequential intervention based on well-being therapy followed by lifestyle intervention and those who underwent lifestyle intervention alone showed a statistically significant decrease in weight.
Delahanty et al. 2020 [50••]	211 (55%)	Adults (age > 18 years, mean age = 62 years), BMI > 25 kg/m^2^ or 23 kg/m^2^ if Asian ancestry (mean BMI = 35 kg/m^2^), HbA1c 65–11.5%, systolic blood pressure (SBP) < 160 mmHg, diastolic blood pressure (DBP) < 100 mmHg, 33% taking insulin, with type 2 diabetes G1 (individual medical nutrition therapy—MNT, *n* = 69) G2 (in-person group lifestyle intervention, *n* = 70) G3 (telephone group lifestyle intervention, *n* = 72)	G1 (MNT): - Frequency: not contain any prespecified approach, content, or number of sessions - Setting: individual - Staff: registered dietitian G2 (in-person lifestyle intervention): - Frequency: 19 group sessions (60–90 min) in the first 6 months (14 weekly sessions followed by 5 bi-weekly) and 18 monthly sessions from 6 months until the end of year 2 + 5 individual sessions over the 2-year - Setting: groups (4–12 patients) + individual - Staff: registered dietitians G3 (telephone lifestyle intervention): - Frequency: 19 group sessions (60–90 min) in the first 6 months (14 weekly sessions followed by 5 bi-weekly) and 18 monthly sessions from 6 months until the end of year 2 + 5 individual sessions over the 2-year - Setting: groups (4–12 patients) + individual - Staff: registered dietitians	Baseline, 6 months, 12 months, 18 months, 24 months (intervention completion), and 36 months	Weight loss from baseline 6 months G1: -1.1% (0.2–2.0%) G2: -5.6% (4.4–6.8%) G3: − 4.6% (3.3–6.0%) 12 months G1: - 2.0% (0.9–3.0%) G2: -4.6% (3.1–6.1%) G3: -4.8% (3.3–6.2%) At least 5% weight loss at 12 months G1: 22% G2: 49% G3: 44% G2 vs. G1: *P* < 0.001 G3 vs. G1: *P* < 0.001 G2 vs. G3: *P* = 0.63	Lifestyle intervention delivered in-person or through telephone lead to weight loss in primary care patients with type 2 diabetes mellitus at a reasonable cost.
St Jules et al. 2023 [35•]	256 (50%)	Adults (age ≥ 40, mean age = 65 (9) years), BMI ≥ 27 kg/m^2^ (mean BMI = 33.8 (5.1) kg/m^2^), with type 2 diabetes and chronic kidney disease G1 (control group—ADVICE, *n* = 64) G2 (Social Cognitive Theory-based Behavioral Group Counseling—SCT, *n* = 64) G3 (technology-based self-monitoring MONITORING, *n* = 64) G4 (COMBINED, *n* = 64)	G1 (ADVICE): - Frequency: Written information on the objectives was initially provided by mail During the next intervention period (6 months), monthly sending of 1-page educational handouts - Setting: individual - Staff: dietitian G2 (SCT): - Frequency: 14 WebEx video-conferencing sessions (1 h). Weekly frequency for the first 4 weeks, then every other week up to 20 weeks - Setting: group (12–15 individuals) - Staff: dietitian G3 (MONITORING): - Frequency: 14 WebEx video-conferencing sessions (1 h). Weekly frequency for the first 4 weeks, then every other week up to 20 weeks. Daily compilation of MyNetDiary - Setting: group (12–15 individuals), individual - Staff: dietitian G4 (COMBINED): - Frequency: 14 WebEx video-conferencing sessions (1 h). Weekly frequency for the first 4 weeks, then every other week up to 20 weeks. Daily compilation of MyNetDiary - Setting: group (12–15 individuals), individual - Staff: dietitian	Baseline, 3 and 6 months	Weight loss, > 5% [*n* (%)] at 3 months G1: 5 (12%) G2: 3 (7%) G3: 10 (20%) G4: 12 (24%) *P* = 0.08 Weight loss, > 5% [*n* (%)] at 6 months G1: 7 (18%) G2: 7 (14%) G3: 9 (20%) G4: 14 (27%) *P* = 0.39 Weight change (kg) at 3 months G1: -1.7 (3.6) G2: -1.4 (2.6) G3: -2.7 (2.7) G4: -2.2 (3.6) *P* = 0.11 Weight change (kg) at 6 months G1: -1.2 (4.3) G2: -1.4 (3.1) G3: -2.3 (3.4) G4: -2.7 (4.4) *P* = 0.19	Patients with type 2 diabetes mellitus, who self-monitored their progress regarding weight loss using a preloaded application on a technological device, lost more weight in the first 3 months of the intervention compared to individuals who did not self-monitor their progress.
Stomby et al. 2020 [58••]	28 (36%)	Adults (age range: 30–70 years, mean age = 60 years), weight-stable (< 5% weight change last 6 months), overweight or obese (BMI range: 25–40 kg/m^2^, mean BMI = 31.4 kg/m^2^), diagnosed with type 2 diabetes within 10 years and treated with lifestyle modification ± metformin G1 (Paleolithic diet alone—PD, *n* = 15) G2 (Paleolithic diet with structured exercise—PDEX, *n* = 13)	G1 (PD): - Frequency: five sessions (the first two meetings were held during the first 2 weeks after baseline measurements and thereafter once a month) - Setting: group - Staff: physician, trained dietician G2 (PDEX): - Frequency: five sessions (the first two meetings were held during the first 2 weeks after baseline measurements and thereafter once a month) + three 1-h exercise sessions per week for 12 weeks (structured high intensity exercise intervention) - Setting: group - Staff: physician, trained dietician, educated personal trainer	Baseline and after 12 weeks	BMI change (kg/m^2^) G1: from 31.4 to 28.9 (*P* < 0.05) G2: from 31.4 to 29.1 (*P* < 0.05) Both interventions were associated with a reduction in BMI (G1: − 8%; G2: − 7%; effect of time *P* < 0.05)	Paleolithic-type diet, with or without physical exercise, was effective in promoting weight loss and reducing insulin sensitivity.
The Look AHEAD Research Group 2022 [44••]	5145 (59.5%)	Adults (age range: 45–76 years; mean age: 59 years), overweight and obese (BMI ≥ 25 kg/m^2^ or ≥ 27 kg/m^2^ if taking insulin; mean BMI: 36 kg/m^2^), with type 2 diabetes (determined by self-report with verification), 15.4% taking insulin, 84% of participants have hypertension, 94% have metabolic syndrome and 14% have a history of cardiovascular disease	G1 (DSE): - Frequency: 3 group educational/social support per year for the first 4 years - Setting: 3 group - Staff: certified diabetic educator and a nutritionist + usual medical care, provided by their own primary care physicians G2 (ILI): Phase I: Months 1 to 6 - Frequency: Weekly - Setting: 3 groups (from 10 to 20 persons, of 60 to 75 min), one individual (20 to 30 min) - Staff: lifestyle counselor Months 7 to 12 - Frequency: 3 per month - Setting: 2 groups, 1 individual - Staff: lifestyle counselor, study physician (or nurse practitioner) Phase II: Years 2 to 4 - Frequency: Minimum of 1 per month - Setting: 1 individual with minimum of 1 additional contact by phone, mail, or e-mail - Staff: lifestyle counselor Phase III: Year 5+ - Frequency: monthly recommended - Setting: individual - Staff: lifestyle counselor General staff: registered dietitian, behavioral psychologist (or other mental health professional) and an exercise specialist supported by a program coordinator, a physician, and a diabetes educator (often a nurse)	Baseline, year 1, year 2, year 3, year 4, and extended follow-up every other year (they wanted to do assessments over 13.5 years but the study was early stopped when the median follow-up was 9.6 years.)	Weight loss at year 1 ILI: 8.5% ± 0.2 of initial weight DSE: 0.6% ± 0.2 of initial weight (*P* < 0.001) Weight loss at year 4 ILI: 4.4% ± 0.2 of initial weight DSE: 0.7% ± 0.2 of initial weight (*P* < 0.001) Weight loss at Year 8 ILI: 4.7% ± 0.2 of initial weight DSE: 2.1% ± 0.2 of initial weight (*P* < 0.001) (mean losses, including participants who had bariatric surgery, were 5.3% ± 0.2% and 2.7% ± 0.2%, respectively) Weight loss from baseline at the end of the study ILI: 6.0% DSE: 3.5%	Compared to Diabetes Support and Education (DSE) program, intensive lifestyle intervention (ILI) led to a significantly greater amount of weight loss in more than 5000 patients with type 2 diabetes mellitus. Moreover, among ILI patients, those who lost ≥ 10% of their body weight during the first year of the intervention, showed a reduced risk of mortality compared to DSE patients.

## Discussion

The aims of the current systematic review were to provide an updated, extensive, and detailed picture on the efficacy of behavior lifestyle interventions in achieving weight loss in T2DM patients, by using the taxonomy proposed by Michie and collaborators [27] of behavior change techniques, and to identify the potential factors, related to the patients and/or the interventions, that could either foster or hamper weight loss. Thirty-one studies, involving 42 behavioral lifestyle interventions for weight loss among patients with T2DM, were identified and discussed in the present work.

Regarding the first aim, the most used behavior change techniques in the studies included in the present systematic review were *goals and planning*, *feedback and monitoring*, *social support*, *shaping knowledge*, *natural consequences*, and* repetition and substitution*. Thanks to the taxonomy proposed by Michie and colleagues [27], we were able to systematically categorize and synthesize, avoiding overlaps, all the behavior change techniques used in the last 10 years of research, which was never done before.

Still related to the first aim, the findings of the current review assessing the efficacy of BLIs in weight reduction in the “real-world practice” of medicine highlighted that all the included behavioral interventions led to a weight loss at least at post-treatment. This is in line with previous work [62] reporting that overweight or obese participants with T2DM who followed diet-based lifestyle interventions, surgery, and pharmacological or multi-component treatment, did not report weight gain between baseline and post-intervention evaluation.

The majority of behavioral interventions characterized by indications regarding diet and/or physical activity and support, seemed to be effective in encouraging weight loss both when compared to a control group that received general indications and had occasional meetings, to waiting list conditions or to absence of treatment. In the last cases, it is noted that even when the interventions included less intensive education sessions, these are equally effective for intervention outcomes, specifically for weight loss. These conclusions seem to go in the same direction as the literature [10, 62–64], confirming the effectiveness of education-based interventions and multi-component behavioral interventions for weight loss in overweight or obese patients with T2DM.

When two or more behavioral interventions were compared, those characterized by regular weekly or bi-weekly feedback meetings and better specified and individualized instructions on diet were more effective, as supported by the analysis of Maula and colleagues [64], which stated that personalized feedback was associated with significant weight loss. Behavioral interventions were also more effective when more frequent contacts with professionals were included.

Among interventions focused on nutrition, high protein, very low-calorie and low-calorie diets, low carbohydrates or low fats diet and ketogenic diet seemed to be effective for significant weight reduction although, in some cases (for example, using a very low calories diet or low fat and high protein diet), an initial weight loss was followed by a weight gain. Specifically, literature showed that the most effective behavioral interventions for weight loss included, in order: education, low-calorie diet and low-carbohydrate meal replacements; education, low-carbohydrate and low-calorie diets; education, low-calorie diets and low-fat meal replacements; interventions involving food education only; education, low-calorie and low-fat diet; and finally, education and motivational interventions [65]. However, similar effects on weight loss were observed regardless of the presence of ingredients focused on motivation. The addition of motivational elements, in fact, did not increase the effectiveness of the behavioral lifestyle interventions, even when the interventions included well-structured protocols on lifestyle in terms of objectives, behavioral indications, and education. This is in line with the results of the systematic review by Maula et al. [64] that, by analyzing the elements of effectiveness of behavioral interventions for weight reduction in overweight or obese patients with T2DM, observed that, among all factors considered, the motivational component was the least effective.

Considering exercise, the results demonstrated that the addition of physical activity did not lead to greater weight loss compared to dietary and behavioral interventions alone. These considerations are not supported by the literature, where the role of physical activity is emphasized, since it is demonstrated that sedentary and inactive lifestyle increases obesity rates [10, 62–66]. Although most studies emphasize the importance of physical activity, those included in the present systematic review are a minority part, including a limited number of participants and making conclusions less generalizable. Moreover, a specific focus on physical activity has been found more effective for T2DM individuals with depressive symptomatology [67], and since in the current systematic review only participants without any psychopathological diagnosis were included, our results on the efficacy of physical activity alone could indicate that overweight or obese T2DM individuals without any depressive symptomatology do not seem to benefit from a specific intervention on physical activity.

Twelve studies included a maintenance phase [30, 38–43, 44••, 45, 51, 53, 54], while 8 had up to 10 years of follow-up evaluations [39–43, 44••, 45]. The cited studies found that, at follow-up, a weight regain might happen but it was not significant. In fact, also in the systematic review by Franz et al. [10], in which they analyzed 11 studies including differentiated protocols, they observed that in general, weight loss could remain even at several years of follow-up.

Although the efficacy of BLI is well-demonstrated, some studies included in the current work did not indicate a significant difference in weight loss between the intervention group and control group [29, 30, 35•, 53]. Furthermore, weight regain after treatment was also observed [53]. Therefore, in relation to the second aim of the current systematic review, according to our findings, it can be argued that the underlying presence of factors that could affect weight loss should be considered. However, it is relevant to note that no study included in the current work specifically analyzed potential interfering patient-related organic characteristics, such as metabolic factors (e.g., resting metabolic rate, adipose tissue lipolysis, brown adipose tissue activity, fibroblast growth factor 21 secretion in response to low-protein hypercaloric diets) [68, 69], level of adipokines secretion [70, 71], and dysregulated gut hormone responses [72], which have been found to influence weight changes. Moreover, no study indicated the presence of non-responders to treatment, nor any models were tested to assess the presence of possible factors that could hamper weight loss. Hence, the factors associated with weight loss and the ones hampering weight loss indicated by the current study were implied by reading each primary study. Furthermore, since the majority of the studies reported a clinically significant weight loss, it is also important to note that factors indicated as not associated with weight loss might be only related to the characteristics of the samples of each study. Based on these premises, the results highlighting the factors associated (i.e., gender, ethnicity, pharmacological and biological variables, number and type of BCTs, and portion-controlled diet, diet high in proteins, and intermittent diet) and factors not associated (i.e., other medical conditions, the absence of ongoing BLI intervention and diabetes support program over time, motivational feedback, focusing only on the weight loss aspect of the intervention, and the presence of a maintenance phase after the intervention) with weight loss cannot be generalized beyond the samples included in the current work. Future studies are needed in order to assess the role of these factors highlighted in the results of the current work and to analyze the predictive value of each specific factor on weight loss.

Among the factors associated with weight loss, Hispanic men seem to achieve better results by attending individual sessions, compared to Hispanic women, who in turn benefit from groups sessions. Previous studies reported mixed results regarding gender differences related to individual and group sessions for weight loss in people without diabetes [73, 74]. Therefore, these results seem to be related also to ethnicity; this is in line with previous works suggesting that individual sessions may be more beneficial to minority men [75]. In relation to pharmacological management, studies in the present systematic review highlighted a clinically significant weight loss, which is in line with previous research suggesting that, for example, a metformin therapy for T2DM patients significantly induces weight loss due to modulation of hypothalamic appetite-regulatory centers [76]. With regards to levels of plasma fetuin-A, it has been proven that it affects insulin resistance, leading to a greater weight loss [77]. In relation to the number and type of BCTs used [59], these results are in line with the work of Michie et al. [27] and also support previous studies suggesting that goal setting and planning and social support are among the most useful BCTs in order to achieve weight loss. Finally, in relation to diet techniques, high protein intake and intermittent diets seem to be more associated with weight loss compared to other approaches, as recently proved by two systematic reviews [78, 79].

Among factors not associated with weight loss, the presence of other medical conditions (e.g., cardiovascular disease) does not seem to be an obstacle to achieve weight loss in patients with T2DM. This could be due to the fact that weight loss can be achieved in different ways (e.g., different kinds of diets), which can be adopted according to the medical comorbidities occured in T2DM patients. In relation to the variables specifically associated with the interventions, motivational feedback sent through email did not foster weight loss. Using a technological device might have been of more help for achieving weight loss, since it provided a real-time feedback, compared to the motivational emails which were sent once each week [30]. According to Carter et al. [53], the absence of ongoing BLIs and dietetic support program over time is associated with weight gain. These results are in line with previous studies, and weight gain can be due to the fact that participants who lost weight better adjusted to the reduced amount of calories required by the diet [53]. Therefore, it could be hypothesized that longer dietetic support programs are needed to allow individuals to adjust to the BLI. Finally, as reported by St-Jules et al. [35•], education on the interventions target alone is not enough for engaging in lifestyle behavior change. The authors [35•] reported a non-significant weight loss in both experimental and control groups, which might have been attenuated by several factors, such as the focus on multiple lifestyle behaviors change and lack of interpersonal contacts.

### Clinical Implications and Limits

Our systematic analysis of the literature on behavioral interventions for weight loss in adults with T2DM and BMI ≥ 25 allowed us to identify a large number of studies, so that it was possible to obtain information about various types of intervention, including diet, physical activity, and behavioral components. This systematic review cannot be compared with any review of the same type, since no analysis in the literature has considered studies that use the same criteria. At the same time, there are no reviews that apply a taxonomy that can allow to classify the ingredients of behavioral interventions, whereas in the present review, the use of Michie’s taxonomy [27] has made possible to subdivide the ingredients of various protocols, with the aim to clear and fast individualize behavioral ingredients and to compare different protocols.

Even though it is difficult to identify specific components, since the literature presents a variety of studies including heterogeneous behavioral ingredients, the present review allows ascertaining some key elements that show more effect on weight than others.

The samples of our studies did not comprehend severely obese patients (BMI > 40). This made our sample more homogeneous but did not make possible to generalize results to the general population, in which diabetics could have a BMI > 40.

The interventions included in this review were very heterogeneous in terms of duration, protocol, and randomization, making a direct comparison between studies difficult. Studies also differed in their sample size, making the generalization of the results complex and not always applicable.

Drop-outs were not considered; this issue could provide additional information regarding how to improve subjects’ adherence to instructions and permanence in this kind of studies.

A further limitation is the fact that there was no statistical analysis to evaluate the most effective ingredients and their specific effect on weight loss. Lastly, another limitation is represented by the fact that all but six studies included in the current work did not depict a clear picture regarding the pharmacological therapies underwent by the patients in each study. Since the intake of these medicines can affect weight loss, future studies should assess the efficacy of BLIs in achieving weight loss in this type of patients by also considering the possible effects (e.g., fostering or hampering weight loss) due to pharmacological therapies [76] (e.g., the use of metformin).

## Conclusions

Although in the literature it emerges the importance of lifestyle interventions in overweight/obese diabetic population, in this systematic review it was found that not all proposed and analyzed interventions are effective for weight loss. In fact, it is not enough to engage behavioral intervention for the management of this disease, but, as the findings of the present study reveal, it is necessary that the behavioral intervention includes specific components to achieve a clinically significant weight loss and, as a result, a general improvement. Findings show that the most effective behavioral interventions for achieving significant weight loss are those that include accurate instructions regarding dietary behavior, stimulate behavioral change through regular meetings (weekly or biweekly), and provide feedback and capillary support to patient’s path through individual or group sessions.

This systematic review shows that physical activity alone, in contrast to diet, does not produce the best results. Specifically, diet is more effective when associated with educational ingredients, with the most effective ones in achieving weight loss through high protein, very low calories (400–500 kcal), low calories (1000–1500 kcal), fat and carbohydrates and ketogenic diets. In contrast, physical activity produces better results when integrated within a global lifestyle intervention, in comparison with exercise alone.

Given the limitations of this work, further investigations including the elements that emerged from this review are needed, in order to systematize effective protocols increasingly widespread and focus on specific targets such as, in this case, patients with T2DM. Moreover, compared to previous works, this systematic review used a more rigorous methodology to assess the efficacy of behavioral lifestyle interventions, which could help healthcare professionals to revise healthcare policies related to behavioral interventions for T2DM patients with overweight/obesity.

### Supplementary Information

Below is the link to the electronic supplementary material.

Supplementary file1 (DOCX 82 KB)

## Data Availability

Not applicable.
