# Supplementary material for: Behavioral Lifestyle Interventions for Weight Loss in Overweight or Obese Patients with Type 2 Diabetes: A Systematic Review of the Literature
Source: Curr Obes Rep. 2024 Mar 4;13(2):224–41. doi: 10.1007/s13679-024-00552-5 (PMC11150315; doi:10.1007/s13679-024-00552-5)
Supplement: Supplementary file 1 — Supplementary file1 (DOCX 82 KB) [file 13679_2024_552_MOESM1_ESM.docx]

**Table S1.** Behavioural components included in Michie *et al.*^27^ taxonomy.

| **AUTHORS, YEAR** | **BEHAVIOR CHANGE TECHNIQUE TAXONOMY** |
| --- | --- |
| Benasi et al.^59^ | G1 (WBT-Lifestyle):  1. Goals and planning: 1.1 Goal setting (behavior), 1.2 Problem solving, 1.3 Goal setting (outcomes), 1.4. Action planning, 1.5. Review behavior goal(s), 1.6. Discrepancy between current behavior and goal, 1.7. Review outcome goal(s)  2. Feedback and monitoring: 2.2. Feedback on behavior, 2.3. Self-monitoring of behaviour, 2.4. Self-monitoring of outcome(s) of behavior, 2.7. Feedback on outcome(s) of behavior  3. Social support: 3.1. Social support (unspecified), 3.3. Social support (emotional)  4. Shaping knowledge: 4.1. Instruction on how to perform the behavior, 4.2. Information about antecedents, 4.3. Riattribuzione  5. Natural consequences: 5.1. Information about health consequences  8. Repetition and substitution: 8.1. Behavioral practice/rehearsal, 8.2. Behavior substitution, 8.3. Habit formation, 8.4. Habit reversal, 8.7. Graded tasks  G2 (Lifestyle Alone,):  1. Goals and planning: 1.1 Goal setting (behavior), 1.2 Problem solving, 1.3 Goal setting (outcomes), 1.4. Action planning, 1.5. Review behavior goal(s), 1.6. Discrepancy between current behavior and goal, 1.7. Review outcome goal(s)  2. Feedback and monitoring: 2.2. Feedback on behavior, 2.3. Self-monitoring of behaviour, 2.4. Self-monitoring of outcome(s) of behavior, 2.7. Feedback on outcome(s) of behavior  3. Social support: 3.1. Social support (unspecified),  4. Shaping knowledge: 4.1. Instruction on how to perform the behavior,  5. Natural consequences: 5.1. Information about health consequences  8. Repetition and substitution: 8.1. Behavioral practice/rehearsal, 8.2. Behavior substitution, 8.3. Habit formation, 8.4. Habit reversal, 8.7. Graded tasks |
| Bentley et al.^30^ | G1:  1. Goals and planning: 1.1. Goal setting (behavior), 1.2. Problem solving  2. Feedback and monitoring: 2.2. Feedback on behaviour, 2.3. Self-monitoring of behaviour  3. Social support: 3.1. Social support (unspecified), 3.3. Social support (emotional)  4. Shaping knowledge: 4.1. Instruction on how to perform the behavior  5. Natural consequences: 5.1. Information about health consequences  6. Comparison of behaviour: 6.1. Demonstration of the behavior  8. Repetition and substitution: 8.1. Behavioral practice/rehearsal, 8.2. Behavior substitution, 8.3. Habit formation, 8.4. Habit reversal  9. Comparison of outcomes: 9.1. Credible source, 9.3. Comparative imagining of future outcomes  16. Covert learning: 16.2. Imaginary reward  G2:  1. Goals and planning: 1.1. Goal setting (behavior), 1.2. Problem solving, 1.4. Action planning  2. Feedback and monitoring: 2.3. Self-monitoring of behaviour, 2.4. Self-monitoring of outcome(s) of behaviour, 2.6. Biofeedback  3. Social support: 3.1. Social support (unspecified), 3.3. Social support (emotional)  4. Shaping knowledge: 4.1. Instruction on how to perform the behavior  5. Natural consequences: 5.1. Information about health consequences  6. Comparison of behaviour: 6.1. Demonstration of the behavior  8. Repetition and substitution: 8.1. Behavioral practice/rehearsal, 8.2. Behavior substitution, 8.3. Habit formation, 8.4. Habit reversal  9. Comparison of outcomes: 9.1. Credible source, 9.3. Comparative imagining of future outcomes  16. Covert learning: 16.2. Imaginary reward  G3:  1. Goals and planning: 1.1. Goal setting (behavior), 1.2. Problem solving, 1.4. Action planning2. Feedback and monitoring: 2.3. Self-monitoring of behaviour, 2.4. Self-monitoring of outcome(s) of behaviour, 2.6. Biofeedback  3. Social support: 3.1. Social support (unspecified), 3.3. Social support (emotional)  4. Shaping knowledge: 4.1. Instruction on how to perform the behavior  5. Natural consequences: 5.1. Information about health consequences  6. Comparison of behaviour: 6.1. Demonstration of the behavior  8. Repetition and substitution: 8.1. Behavioral practice/rehearsal, 8.2. Behavior substitution, 8.3. Habit formation, 8.4. Habit reversal  9. Comparison of outcomes: 9.1. Credible source, 9.3. Comparative imagining of future outcomes  16. Covert learning: 16.2. Imaginary reward |
| Carter et al.^31^ | G1 (Intermittent): 1. Goals and planning: 1.1. Goal setting (behavior) 2. Feedback and monitoring: 2.2. Feedback on behaviour, 2.3. Self-monitoring of behaviour 3. Social support: 3.1. Social support (unspecified) 4. Shaping knowledge: 4.1. Instruction on how to perform the behavior 5. Natural consequences: 5.1. Information about health consequences 8. Repetition and substitution: 8.1. Behavioral practice/rehearsal, 8.2. Behavior substitution, 8.3. Habit formation, 8.4. Habit reversal 9. Comparison of outcomes: 9.1. Credible source  G2 (Continuous): 1. Goals and planning: 1.1. Goal setting (behavior) 2. Feedback and monitoring: 2.2. Feedback on behaviour, 2.3. Self-monitoring of behaviour 3. Social support: 3.1. Social support (unspecified) 4. Shaping knowledge: 4.1. Instruction on how to perform the behavior 5. Natural consequences: 5.1. Information about health consequences 8. Repetition and substitution: 8.1. Behavioral practice/rehearsal, 8.2. Behavior substitution, 8.3. Habit formation, 8.4. Habit reversal 9. Comparison of outcomes: 9.1. Credible source |
| Delahanty et al.^44^ | G1 (Lifestyle intervention):  1. Goals and planning: 1.1. Goal setting (behavior), 1.2. Problem solving, 1.3. Goal setting (outcome), 1.5. Review behavior goal(s)  2. Feedback and monitoring: 2.2. Feedback on behaviour, 2.3. Self-monitoring of behaviour  3. Social support: 3.1. Social support (unspecified)  4. Shaping knowledge: 4.1. Instruction on how to perform the behavior  5. Natural consequences: 5.1. Information about health consequences  8. Repetition and substitution: 8.1. Behavioral practice/rehearsal, 8.2. Behavior substitution, 8.3. Habit formation, 8.4. Habit reversal, 8.7. Graded tasks  9. Comparison of outcomes: 9.1. Credible source  G2 (RD):  1. Goals and planning: 1.1. Goal setting (behavior), 1.3. Goal setting (outcome)  2. Feedback and monitoring: 2.2. Feedback on behaviour, 2.3. Self-monitoring of behaviour  5. Natural consequences: 5.1. Information about health consequences  9. Comparison of outcomes: 9.1. Credible source |
| Delahanty et al.^45^ | G1 (MNT): 1. Goals and planning: 1.1. Goal setting (behavior), 1.5. Review behavior goal(s) 2. Feedback and monitoring: 2.2. Feedback on behaviour 3. Social support: 3.1. Social support (unspecified) 4. Shaping knowledge: 4.1. Instruction on how to perform the behavior 5. Natural consequences: 5.1. Information about health consequences 8. Repetition and substitution: 8.1. Behavioral practice/rehearsal, 8.2. Behavior substitution, 8.3. Habit formation, 8.4. Habit reversal 9. Comparison of outcomes: 9.1. Credible source  G2 and G3 (Lifestyle interventions): 1. Goals and planning: 1.1. Goal setting (behavior), 1.5. Review behavior goal(s) 2. Feedback and monitoring: 2.2. Feedback on behaviour, 2.3. Self-monitoring of behaviour 3. Social support: 3.1. Social support (unspecified), 3.3. Social support (emotional) 4. Shaping knowledge: 4.1. Instruction on how to perform the behavior 5. Natural consequences: 5.1. Information about health consequences, 5.2. Salience of consequences, 5.4. Monitoring of emotional consequences, 5.6. Information about emotional consequences 8. Repetition and substitution: 8.1. Behavioral practice/rehearsal, 8.2. Behavior substitution, 8.3. Habit formation, 8.4. Habit reversal 9. Comparison of outcomes: 9.1. Credible source |
| Delahanty et al.^46^ | G1 (MNT):  1. Goals and planning: 1.1. Goal setting (behavior), 1.5. Review behavior goal(s)  2. Feedback and monitoring: 2.2. Feedback on behaviour  3. Social support: 3.1. Social support (unspecified)  4. Shaping knowledge: 4.1. Instruction on how to perform the behavior  5. Natural consequences: 5.1. Information about health consequences  8. Repetition and substitution: 8.1. Behavioral practice/rehearsal, 8.2. Behavior substitution, 8.3. Habit formation, 8.4. Habit reversal  9. Comparison of outcomes: 9.1. Credible source  G2 and G3 (Lifestyle interventions):  1. Goals and planning: 1.1. Goal setting (behavior), 1.5. Review behavior goal(s)  2. Feedback and monitoring: 2.2. Feedback on behaviour, 2.3. Self-monitoring of behaviour  3. Social support: 3.1. Social support (unspecified), 3.3. Social support (emotional)  4. Shaping knowledge: 4.1. Instruction on how to perform the behavior  5. Natural consequences: 5.1. Information about health consequences, 5.2. Salience of consequences, 5.4. Monitoring of emotional consequences, 5.6. Information about emotional consequences  8. Repetition and substitution: 8.1. Behavioral practice/rehearsal, 8.2. Behavior substitution, 8.3. Habit formation, 8.4. Habit reversal  9. Comparison of outcomes: 9.1. Credible source |
| Foster et al.^43^ | G1 (Lifestyle intervention with PCD): 1. Goals and planning: 1.1. Goal setting (behavior), 1.2. Problem solving, 1.4. Action planning 2. Feedback and monitoring: 2.2 Feedback on behavior, 2.3. Self-monitoring of behaviour, 2.4. Self-monitoring of outcome(s) of behaviour, 2.7. Feedback on outcome(s) of behavior  3. Social support: 3.1. Social support (unspecified) 4. Shaping knowledge: 4.1. Instruction on how to perform the behavior, 4.2. Information about antecedents 5. Natural consequences: 5.1. Information about health consequences 8. Repetition and substitution: 8.2. Behavior substitution, 8.3. Habit formation, 8.4. Habit reversal, 8.7. Graded tasks 9. Comparison of outcomes: 9.1. Credible source  12 Antecedents: 12.3. Avoidance/reducing exposure to cues for the behavior  G2 (DSME): 2. Feedback and monitoring: 2.2 Feedback on behavior, 2.3. Self-monitoring of behaviour, 2.4. Self-monitoring of outcome(s) of behavior, 2.7. Feedback on outcome(s) of behavior  4. Shaping knowledge: 4.1. Instruction on how to perform the behavior  8. Repetition and substitution: 8.2. Behavior substitution  9. Comparison of outcomes: 9.1. Credible source |
| Gamiochipi et al.^47^ | G1 (IIEV): 1. Goals and planning: 1.1. Goal setting (behavior), 1.2. Problem solving, 1.3. Goal setting (outcome), 1.4. Action planning, 1.5. Review behavior goal(s) 2. Feedback and monitoring: 2.2. Feedback on behaviour, 2.3. Self-monitoring of behaviour 3. Social support: 3.1. Social support (unspecified), 3.3. Social support (emotional) 4. Shaping knowledge: 4.1. Instruction on how to perform the behavior 5. Natural consequences: 5.1. Information about health consequences, 5.2. Salience of consequences 8. Repetition and substitution: 8.1. Behavioral practice/rehearsal, 8.2. Behavior substitution, 8.3. Habit formation, 8.4. Habit reversal, 8.7. Graded tasks 9. Comparison of outcomes: 9.1. Credible source 10. Reward and threat: 10.3. Non-specific reward 12. Antecedents: 12.1. Restructuring the physical environment  G2 (COED): 1. Goals and planning: 1.1. Goal setting (behavior), 1.2. Problem solving, 1.4. Action planning, 1.5. Review behavior goal(s), 1.9. Commitment 2. Feedback and monitoring: 2.2. Feedback on behaviour, 2.3. Self-monitoring of behaviour 3. Social support: 3.1. Social support (unspecified), 3.3. Social support (emotional) 4. Shaping knowledge: 4.1. Instruction on how to perform the behavior 5. Natural consequences: 5.1. Information about health consequences, 5.2. Salience of consequences 8. Repetition and substitution: 8.1. Behavioral practice/rehearsal, 8.2. Behavior substitution, 8.3. Habit formation, 8.4. Habit reversal, 8.7. Graded tasks 9. Comparison of outcomes: 9.1. Credible source 11. Regulation: 11.2. Reduce negative emotions |
| Goday et al.^36^ | G1 (VLCK diet group):  1. Goals and planning: 1.1. Goal setting (behavior), 1.3. Goal setting (outcome), 1.5. Review behavior goal(s)  2. Feedback and monitoring: 2.2. Feedback on behaviour, 2.3. Self-monitoring of behaviour, 2.4. Self-monitoring of outcome(s) of behaviour, 2.7. Feedback on outcome(s) of behavior  3. Social support: 3.1. Social support (unspecified)  4. Shaping knowledge: 4.1. Instruction on how to perform the behavior  5. Natural consequences: 5.1. Information about health consequences  8. Repetition and substitution: 8.1. Behavioral practice/rehearsal, 8.2. Behavior substitution, 8.3. Habit formation, 8.4. Habit reversal, 8.7. Graded tasks  9. Comparison of outcomes: 9.1. Credible source  G2 (LC diet group):  1. Goals and planning: 1.1. Goal setting (behavior)  2. Feedback and monitoring: 2.2. Feedback on behaviour, 2.3. Self-monitoring of behaviour, 2.7. Feedback on outcome(s) of behavior  3. Social support: 3.1. Social support (unspecified)  4. Shaping knowledge: 4.1. Instruction on how to perform the behavior  8. Repetition and substitution: 8.1. Behavioral practice/rehearsal, 8.2. Behavior substitution, 8.3. Habit formation, 8.4. Habit reversal  9. Comparison of outcomes: 9.1. Credible source |
| Hankonen et al.^41^ | G1 (*ADDITION* Plus): 1. Goals and planning: 1.1. Goal setting (behavior), 1.4. Action planning, 1.5. Review behavior goal(s) 2. Feedback and monitoring: 2.3. Self-monitoring of behaviour 3. Social support: 3.1. Social support (unspecified) 4. Shaping knowledge: 4.1. Instruction on how to perform the behavior 5. Natural consequences: 5.1. Information about health consequences  7. Associations: 7.1. Prompts/cues 8. Repetition and substitution: 8.2. Behavior substitution, 8.3. Habit formation, 8.4. Habit reversal 15. Self-belief: 15.2. Mental rehearsal of successful performance  G2 (Intensive diabetes treatment): 1. Goals and planning: 1.1. Goal setting (behavior), 1.5. Review behavior goal(s) 2. Feedback and monitoring: 2.2. Feedback on behaviour, 2.3. Self-monitoring of behaviour 3. Social support: 3.1. Social support (unspecified) 5. Natural consequences: 5.1. Information about health consequences 8. Repetition and substitution: 8.1. Behavioral practice/rehearsal, 8.2. Behavior substitution, 8.3. Habit formation, 8.4. Habit reversal, 8.7. Graded tasks |
| Kuna et al.^40^ | G1 (Intensive lifestyle intervention):  1. Goals and planning: 1.1. Goal setting (behavior), 1.3. Goal setting (outcome), 1.4. Action planning, 1.5. Review behavior goal(s), 1.7. Review outcome goal(s)  2. Feedback and monitoring: 2.2. Feedback on behaviour, 2.3. Self-monitoring of behaviour, 2.4. Self-monitoring of outcome(s) of behaviour, 2.7. Feedback on outcome(s) of behavior  3. Social support: 3.1. Social support (unspecified)  4. Shaping knowledge: 4.1. Instruction on how to perform the behavior  5. Natural consequences: 5.1. Information about health consequences  6. Comparison of behaviour: 6.1. Demonstration of the behavior  8. Repetition and substitution: 8.1, Behavioral practice/rehearsal, 8.2. Behavior substitution, 8.3. Habit formation, 8.4. Habit reversal  9. Comparison of outcomes: 9.1. Credible source  G2 (DSE):  1. Goals and planning: 1.1. Goal setting (behavior)  3. Social support: 3.2. Social support (unspecified)  5. Natural consequences: 5.1. Information about health consequences |
| Lutes et al.^38^ | G1 (Phone-based lifestyle intervention):  1. Goals and planning: 1.1. Goal setting (behavior), 1.2. Problem solving, 1.5. Review behavior goal(s)  2. Feedback and monitoring: 2.2. Feedback on behaviour, 2.3. Self-monitoring of behaviour, 2.7. Feedback on outcome(s) of behavior  4. Shaping knowledge: 4.1. Instruction on how to perform the behavior, 4.3. Re-attribution  5. Natural consequences: 5.1. Information about health consequences, 5.6. Information about emotional consequences  8. Repetition and substitution: 8.1. Behavioral practice/rehearsal, 8.2. Behavior substitution, 8.3. Habit formation, 8.4. Habit reversal, 8.7. Graded tasks  11. Regulation: 11.2. Reduce negative emotion  G2 (Educational mailings):  1. Goals and planning: 1.1. Goal setting (behavior)  2. Feedback and monitoring: 2.3. Self-monitoring of behavior  5. Natural consequences: 5.1. Information about health consequences, |
| Moncrieft et al.^39^ | G1 (Lifestyle intervention): 1. Goals and planning: 1.1. Goal setting (behavior), 1.2. Problem solving, 1.3. Goal setting (outcome), 1.4. Action planning 2. Feedback and monitoring: 2.3. Self-monitoring of behaviour, 2.4. Self-monitoring of outcome(s) of behaviour 3. Social support: 3.1. Social support (unspecified) 4. Shaping knowledge: 4.1. Instruction on how to perform the behavior 5. Natural consequences: 5.1. Information about health consequences, 5.4. Monitoring of emotional consequences 8. Repetition and substitution: 8.1. Behavioral practice/rehearsal, 8.2. Behavior substitution, 8.3. Habit formation, 8.4. Habit reversal  10. Reward and threat: 10.2. Material reward, 10.8. Incentive (outcome), 10.10 Reward (outcome)  11. Regulation: 11.2. Reduce negative emotion  G2 (Usual care): 4. Shaping knowledge: 4.1. Instruction on how to perform the behavior 5. Natural consequences: 5.1. Information about health consequences  10. Reward and threat: 10.2. Material reward, 10.8. Incentive (outcome), 10.10 Reward (outcome) |
| Osama and Shehab^58^ | G1 (Group A):  1. Goals and planning: 1.1. Goal setting (behavior), 1.4. Action planning, 1.5. Review behavior goal(s)  2. Feedback and monitoring: 2.2. Feedback on behaviour, 2.3. Self-monitoring of behaviour  3. Social support: 3.1. Social support (unspecified), 3.2. Social support (practical)  4. Shaping knowledge: 4.1. Instruction on how to perform the behavior  5. Natural consequences: 5.1. Information about health consequences  8. Repetition and substitution: 8.1. Behavioral practice/rehearsal, 8.2. Behavior substitution, 8.3. Habit formation, 8.4. Habit reversal, 8.7. Graded tasks  9. Comparison of outcomes: 9.1. Credible source  G2 (Group B):  none |
| Otten et al.^33-34^ | G1 (PD): 1. Goals and planning: 1.1. Goal setting (behavior), 1.4. Action planning 2. Feedback and monitoring: 2.2. Feedback on behaviour, 2.3. Self-monitoring of behaviour, 2.4. Self-monitoring of outcome(s) of behaviour 3. Social support: 3.1. Social support (unspecified) 4. Shaping knowledge: 4.1. Instruction on how to perform the behavior 5. Natural consequences: 5.1. Information about health consequences 8. Repetition and substitution: 8.1. Behavioral practice/rehearsal, 8.2. Behavior substitution, 8.3. Habit formation, 8.4. Habit reversal 9. Comparison of outcomes: 9.1. Credible source  G2 (PD-EX): 1. Goals and planning: 1.1. Goal setting (behavior), 1.4. Action planning, 1.5. Review behavior goal(s) 2. Feedback and monitoring: 2.2. Feedback on behaviour, 2.3. Self-monitoring of behaviour, 2.4. Self-monitoring of outcome(s) of behaviour 3. Social support: 3.1. Social support (unspecified) 4. Shaping knowledge: 4.1. Instruction on how to perform the behavior 5. Natural consequences: 5.1. Information about health consequences 6. Comparison of behaviour: 6.1. Demonstration of the behavior 8. Repetition and substitution: 8.1. Behavioral practice/rehearsal, 8.2. Behavior substitution, 8.3. Habit formation, 8.4. Habit reversal, 8.7. Graded tasks 9. Comparison of outcomes: 9.1. Credible source |
| St-Jules et al.^37^ | G1 (Intervention): 1. Goals and planning: 1.1. Goal setting (behavior), 1.4. Action planning, 1.5. Review behavior goal(s) 2. Feedback and monitoring: 2.2. Feedback on behaviour, 2.3. Self-monitoring of behavior, 2.7. Feedback on outcome(s) of behavior 3. Social support: 3.1. Social support (unspecified) 4. Shaping knowledge: 4.1. Instruction on how to perform the behavior, 4.3. Re-attribution 5. Natural consequences: 5.1. Information about health consequences 8. Repetition and substitution: 8.1. Behavioral practice/rehearsal, 8.2. Behavior substitution, 8.3. Habit formation, 8.4. Habit reversal  9. Comparison of outcomes: 9.1. Credible source  G2 (Control): 1. Goals and planning: 1.1. Goal setting (behavior) 2. Feedback and monitoring: 2.2. Feedback on behaviour, 2.3. Self-monitoring of behaviour 4. Shaping knowledge: 4.1. Instruction on how to perform the behavior 5. Natural consequences: 5.1. Information about health consequences |
| Stomby et al.^57^ | G1 (PD): 1. Goals and planning: 1.1. Goal setting (behavior) 2. Feedback and monitoring: 2.2. Feedback on behaviour, 2.3. Self-monitoring of behaviour 3. Social support: 3.1. Social support (unspecified), 3.2. Social support (practical) 4. Shaping knowledge: 4.1. Instruction on how to perform the behavior 5. Natural consequences: 5.1. Information about health consequences 6. Comparison of behaviour: 6.1. Demonstration of the behavior 8. Repetition and substitution: 8.1. Behavioral practice/rehearsal, 8.2. Behavior substitution, 8.3. Habit formation, 8.4. Habit reversal 9. Comparison of outcomes: 9.1. Credible source  G2 (PDEX): 1. Goals and planning: 1.1. Goal setting (behavior), 1.3. Goal setting (outcome), 1.4. Action planning 2. Feedback and monitoring: 2.2. Feedback on behaviour, 2.3. Self-monitoring of behaviour, 2.4. Self-monitoring of outcome(s) of behaviour, 2.7. Feedback on outcome(s) of behavior 3. Social support: 3.1. Social support (unspecified), 3.2. Social support (practical) 4. Shaping knowledge: 4.1. Instruction on how to perform the behavior 5. Natural consequences: 5.1. Information about health consequences 6. Comparison of behaviour: 6.1. Demonstration of the behavior 8. Repetition and substitution: 8.1. Behavioral practice/rehearsal, 8.2. Behavior substitution, 8.3. Habit formation, 8.4. Habit reversal 9. Comparison of outcomes: 9.1. Credible source |
| The Look AHEAD Research Group^42,48-56^ | G1 (DSE):  1. Goals and planning: 1.1. Goal setting (behavior)  2. Feedback and monitoring: 2.2. Feedback on behaviour, 2.7. Feedback on outcome(s) of behavior  3. Social support: 3.2. Social support (unspecified)  4. Shaping knowledge: 4.1. Instruction on how to perform the behavior  5. Natural consequences: 5.1. Information about health consequences  8. Repetition and substitution: 8.1. Behavioral practice/rehearsal, 8.2. Behavior substitution, 8.3. Habit formation, 8.4. Habit reversal  9. Comparison of outcomes: 9.1. Credible source  G2 (ILI):  1. Goals and planning: 1.1. Goal setting (behavior), 1.3. Goal setting (outcome), 1.4. Action planning, 1.5. Review behavior goal(s), 1.7. Review outcome goal(s)  2. Feedback and monitoring: 2.2. Feedback on behaviour, 2.3. Self-monitoring of behaviour, 2.4. Self-monitoring of outcome(s) of behaviour, 2.7. Feedback on outcome(s) of behavior  3. Social support: 3.1. Social support (unspecified)  4. Shaping knowledge: 4.1. Instruction on how to perform the behavior  5. Natural consequences: 5.1. Information about health consequences  6. Comparison of behaviour: 6.1. Demonstration of the behavior  8. Repetition and substitution: 8.1, Behavioral practice/rehearsal, 8.2. Behavior substitution, 8.3. Habit formation, 8.4. Habit reversal  9. Comparison of outcomes: 9.1. Credible source |
| Wang et al.^29^ | G1 (Mobile group): 1. Goals and planning: 1.1. Goal setting (behavior), 1.2. Problem solving, 1.4. Action planning 2. Feedback and monitoring: 2.2. Feedback on behaviour, 2.3. Self-monitoring of behaviour, 2.4. Self-monitoring of outcome(s) of behaviour, 2.7. Feedback on outcome(s) of behavior 3. Social support: 3.1. Social support (unspecified) 4. Shaping knowledge: 4.1. Instruction on how to perform the behavior 5. Natural consequences: 5.1. Information about health consequences 8. Repetition and substitution: 8.1. Behavioral practice/rehearsal, 8.2. Behavior substitution, 8.3. Habit formation, 8.4. Habit reversal 10. Reward and threat: 10.6. Non-specific incentive  G2 (Paper group):  1. Goals and planning: 1.1. Goal setting (behavior), 1.2. Problem solving, 1.4. Action planning 2. Feedback and monitoring: 2.2. Feedback on behaviour, 2.3. Self-monitoring of behaviour, 2.4. Self-monitoring of outcome(s) of behaviour, 2.7. Feedback on outcome(s) of behavior 3. Social support: 3.1. Social support (unspecified) 4. Shaping knowledge: 4.1. Instruction on how to perform the behavior 5. Natural consequences: 5.1. Information about health consequences 8. Repetition and substitution: 8.1. Behavioral practice/rehearsal, 8.2. Behavior substitution, 8.3. Habit formation, 8.4. Habit reversal 10. Reward and threat: 10.6. Non-specific incentive  G3 (Usual care): 1. Goals and planning: 1.3. Goal setting (outcome) 4. Shaping knowledge: 4.1. Instruction on how to perform the behavior 5. Natural consequences: 5.1. Information about health consequences |
| Watson et al.^32^ | G1 (HP): 1. Goals and planning: 1.1. Goal setting (behavior), 1.3. Goal setting (outcome), 1.4. Action planning, 1.5. Review behavior goal(s) 2. Feedback and monitoring: 2.2. Feedback on behaviour, 2.3. Self-monitoring of behaviour, 2.4. Self-monitoring of outcome(s) of behaviour, 2.7. Feedback on outcome(s) of behavior 3. Social support: 3.1. Social support (unspecified) 4. Shaping knowledge: 4.1. Instruction on how to perform the behavior 5. Natural consequences: 5.1. Information about health consequences 8. Repetition and substitution: 8.1. Behavioral practice/rehearsal, 8.2. Behavior substitution, 8.3. Habit formation, 8.4. Habit reversal, 8.7. Graded tasks 9. Comparison of outcomes: 9.1. Credible source  G2 (HC): 1. Goals and planning: 1.1. Goal setting (behavior), 1.3. Goal setting (outcome), 1.4. Action planning, 1.5. Review behavior goal(s) 2. Feedback and monitoring: 2.2. Feedback on behaviour, 2.3. Self-monitoring of behaviour, 2.4. Self-monitoring of outcome(s) of behaviour, 2.7. Feedback on outcome(s) of behavior 3. Social support: 3.1. Social support (unspecified) 4. Shaping knowledge: 4.1. Instruction on how to perform the behavior 5. Natural consequences: 5.1. Information about health consequences 8. Repetition and substitution: 8.1. Behavioral practice/rehearsal, 8.2. Behavior substitution, 8.3. Habit formation, 8.4. Habit reversal, 8.7. Graded tasks 9. Comparison of outcomes: 9.1. Credible source |
| Ziegler et al.^35^ | G1 (HF-RM+C): 1. Goals and planning: 1.1. Goal setting (behavior), 1.4. Action planning 2. Feedback and monitoring: 2.2. Feedback on behaviour, 2.3. Self-monitoring of behaviour, 2.4. Self-monitoring of outcome(s) of behaviour 3. Social support: 3.1. Social support (unspecified) 4. Shaping knowledge: 4.1. Instruction on how to perform the behavior 5. Natural consequences: 5.1. Information about health consequences 8. Repetition and substitution: 8.1. Behavioral practice/rehearsal, 8.2. Behavior substitution, 8.3. Habit formation, 8.4. Habit reversal 9. Comparison of outcomes: 9.1. Credible source 10. Reward and threat: 10.2. Material reward (behaviour)  G2 (LF+RM-C): 1. Goals and planning: 1.1. Goal setting (behavior), 1.4. Action planning 2. Feedback and monitoring: 2.2. Feedback on behaviour, 2.3. Self-monitoring of behaviour, 2.4. Self-monitoring of outcome(s) of behaviour 3. Social support: 3.1. Social support (unspecified) 4. Shaping knowledge: 4.1. Instruction on how to perform the behavior 5. Natural consequences: 5.1. Information about health consequences 8. Repetition and substitution: 8.1. Behavioral practice/rehearsal, 8.2. Behavior substitution, 8.3. Habit formation, 8.4. Habit reversal 9. Comparison of outcomes: 9.1. Credible source 10. Reward and threat: 10.2. Material reward (behaviour) |

**Table S2.** Studies comparing behavioral interventions.

| **AUTHORS, YEAR** | **COUNTRY** | **N** | **%F** | **STUDY DESIGN** | **SAMPLE CHARACTERISTICS** | **CHARACTERISTICS OF THE INTERVENTION  (FREQUENCY, DURATION, SETTING, TYPE OF STAFF)** | **ASSESSMENT (AND FOLLOW-UP)** | **WEIGHT AND/OR BMI** | **WEIGHT OUTCOME** | **ANTHROPOMETRIC AND METABOLIC PARAMETERS** |
| --- | --- | --- | --- | --- | --- | --- | --- | --- | --- | --- |
| Benasi et al.^59^ | Italy | 58 | 40% | Feasibility study of a RCT | Adults (age range: 18-65 years; mean age = 55,5 (6,6) years), BMI ≥ 25 kg/m2, 4 (2-5) with medical comorbidities, 22% taking insulin, with type 2 diabetes  G1 (WBT-Lifestyle, n=30)  G2 (Lifestyle Alone, n=28) | G1 (WBT-Lifestyle):  - Frequency: 4 weekly 1 hr long individual sessions of WBT; 12 weekly individual sessions of lifestyle intervention, 4 of which were 1hr long and conducted in person, the remaining 8 were 30 minutes long and delivered through telephone calls  - Setting: individual  - Staff: psychoterapists specialized in WBT, clinical psychologists  G2 (Lifestyle Alone):  - Frequency: 12 weekly individual sessions, 4 of which were 1hr long and conducted in person, the remaining 8 were 30 minutes long and delivered through telephone calls  - Setting: individual  - Staff: clinical psychologists | Baseline, 4 months, 6 months e 12 months | Weight (kg) at baseline:  G1: 94.8 (23.4)  G2: 95.6 (19.1)  Weight (kg) at 4 months:  G1: 96.4 (26.1)  G2: 93.2 (17.2)  P= 0.50  Weight (kg) at 6 months:  G1: 97.1 (26.7)  G2: 93.3 (17.9)  P= 0.62  Weight (kg) at 12 months:  G1: 93.0 (19.9)  G2: 90.6 (17.6)  P= 0.48 | Primary | *HbA1c (%)* at baseline:  G1: 8.3 (1.7)  G2: 7.9 (1.2)  *HbA1c (%)* at 4 months:  G1: 7.9 (1.2)  G2: 7.2 (1.1)  P= 0.52  *HbA1c (%)* at 6 months:  G1: 8.1 (1.6)  G2: 8.3 (1.3)  P= 0.81  *HbA1c (%)* at 12 months:  G1: 8.5 (1.0)  G2: 8.2 (2.5)  P= 0.53  *HDL (mg/dL)* at baseline:  G1: 46.8 (10.4)  G2: 48.8 (16.7)  *HDL (mg/dL)* at 4 months:  G1: 49.3 (6.3)  G2: 44.8 (11.9)  P= 0.09  *HDL (mg/dL)* at 6 months:  G1: 42.6 (3.5)  G2: 43.1 (10.3)  P= 0.16  *HDL (mg/dL)* at 12 months:  G1: 46.8 (4.8)  G2: 48.0 (10.5)  P= 0.22  *LDL (mg/dL)* at baseline:  G1: 98.9 (41.6)  G2: 95.2 (18.4)  *LDL (mg/dL)* at 4 months:  G1: 79.7 (35.1)  G2: 107.2 (22.2)  P= 0.79  *LDL (mg/dL)* at 6 months:  G1: 113.4 (34.5)  G2: 99.9 (24.6)  P= 0.40  *LDL (mg/dL)* at 12 months:  G1: 87.3 (39.5)  G2: 107.6 (14.9)  P= 0.60  *TG (mg/dL)* at baseline:  G1: 209.1 (163.7)  G2: 170.9 (75.1)  *TG (mg/dL)* at 4 months:  G1: 178.7 (73.3)  G2: 172.8 (95.6)  P= 0.90  *TG (mg/dL)* at 6 months:  G1: 173.2 (126.5)  G2: 138.6 (61.1)  P= 0.74  *TG (mg/dL)* at 12 months:  G1: 110.2 (76.7)  G2: 183.4 (61.7)  P= 0.019  *SBP (mm Hg)* at baseline:  G1: 131.6 (16.9)  G2: 132.6 (13.4)  *SBP (mm Hg)* at 4 months:  G1: 125.4 (11.0)  G2: 130.7 (15.9)  P= 0.64  *SBP (mm Hg)* at 6 months:  G1: 122.8 (13.0)  G2: 132.0 (14.6)  P= 0.13  *SBP (mm Hg)* at 12 months::  G1: 125.0 (12.9)  G2: 140.0 (27.5)  P= 0.10  *DBP (mm Hg)* at baseline:  G1: 77.9 (6.3)  G2: 77.6 (8.1)  *DBP (mm Hg)* at 4 months:  G1: 75.0 (6.7)  G2: 78.6 (3.8)  P= 0.21  *DBP (mm Hg)* at 6 months:  G1: 72.2 (8.3)  G2: 77.0 (6.7)  P= 0.09  *DBP (mm Hg)* at 12 months:  G1: 72.5 (5.0)  G2: 78.6 (15.7)  P= 0.24 |
| Bentley et al.^30^ | UK | 27 | 57% | Feasibility study of a RCT | Adults (age range: 30–60 years; mean age= 53 years), BMI between 25 kg/m2 and 40 kg/m2, with type 2 diabetes  G1 (Advice on diet and exercise, n=9) G2 (G1 plus use of AiperMotion 500, n=9) G3 (G2 plus email motivational support, n=9) | G1: - Frequency: At the beginning of the 12 week intervention all participants received 90-min group training. 12 week intervention followed by a 4-weeks weight maintenance period.  - Setting: group training - Staff: research team  G2: - Frequency: At the beginning of the 12 week intervention all participants received 90-min group training + 60 min training in use of the AiperMotion 500. 12 week intervention followed by a 4-weeks weight maintenance period. - Setting: group training - Staff: research team  G3: - Frequency: At the beginning of the 12 week intervention all participants received 90-min group training + 60 min training in use of the AiperMotion 500 + weekly emails to the research team. 12 week intervention followed by a 4-weeks weight maintenance period. - Setting: group training - Staff: research team | Baseline and weeks 6, 12, and 16. | Weight change (kg) from baseline at week 6 G1: +0.1 (−1.0 to +1.6)* G2: −2.7 (−5.0 to–1.2)* G3: −3.0 (−8.6 to +0.4)*  Weight change (kg) from baseline at week 12 G1: +0.8 (−0.2 to + 2.1)* G2: −2.9 (−7.4 to–0.7)* G3: −3.2 (−12.0 to +2.0)*  Weight change (kg) from baseline at week 16 G1: +0.7 (−0.6 to +2.0)* G2: −3.3 (−8.8 to–0.6)* G3: −3.0 (−13.0 to +3.0)*  *Range change from Baseline | Primary | HbA1c change (mmol/mol) from baseline at week 6 G1: −0.9 (–9.0 to +5.0)* G2: −4.8 (–12 to +3.0)* G3: −2.9 (–10.0 to 0.0)*  HbA1c change (mmol/mol) from baseline at week 12 G1: 0.0 (–4.0 to +3.0)* G2: −8.1 (–13.0 to 0.0)* G3: −14.8 (-39.0 to–2.0)*  HbA1c change (mmol/mol) from baseline at week 16 G1: +0.9 (–5.0 to +9.0)* G2: −10.7 (–26.0 to 0.0)* G3: −5.0 (–12.0 to +2.0)*  *Range change from Baseline |
| Delahantly et al.^44^ | US | 57 | 41% | Randomized controlled trial (RCT) | Adults (age ≥ 18 years, mean age = 61 years), BMI > 25 kg/m2, HbA1c 7-11%, Systolic blood pressure (SBP) < 160 mmHg, diastolic blood pressure (DBP) < 100 mmHg, 41/57 taking insulin, with type 2 diabetes  G1 (Lifestyle intervention - GLI, n=29) G2 (Dietitian referral - RD, n=28) | G1 (GLI): - Frequency: 19 weekly 1.5-hr group sessions with 8-10 study participants per group during the 6- month study period. Meeting with the study physician at the first session for 10-15 min. - Setting: group - Staff: study physician, dietitian  G2 (RD): - Frequency: 1 hr initial sessions with 20-40 min follow-up sessions, not contain number of sessions - Setting: individual - Staff: MGH dietitian | Baseline, 6 months and 12 months | Changes from baseline to 6-month  Weight loss ≥5% (n)  G1: 13 (46%)  G2: 6 (21%)  P= 0.04  Weight loss ≥10% (n)  G1: 9 (32%)  G2: 1 (3%)  P= 0.01  Change in weight (kg)  G1: -6.65 (7.00 SD)  G2: -2.09 (3.50 SD)  P= 0.004  Percent weight change  G1: -6.58 (6.18 SD)  G2: -2.22 (3.73 SD)  P= 0.003  Changes from baseline to 12-month  Weight loss ≥5%  G1: 39%  G2: 24%  P= 0.2  Mean weight change  G1: 5.6%  G2: 1.7%  P= 0.008 | Primary | Changes from baseline to 6-month  Change in HbA1c  G1: -0.70 (1.13 SD)  G2: -0.39 (1.51 SD)  P= 0.38  Change in systolic blood pressure (mmHg)  G1: -5.04 (20.12 SD)  G2: -1.05 (15.76 SD)  P= 0.41  Change in diastolic blood pressure (mmHg)  G1: -2.41 (8.98 SD)  G2: -2.36 (9.12 SD)  P= 0.98  Change in total cholesterol, (mg/dL)  G1: -5.18 (26.12 SD)  G2: -5.31 (42.34 SD)  P= 0.99  Change in LDL (mg/dL)  G1: -6.50 (22.79 SD)  G2: 2.28 (20.35 SD)  P= 0.13  Change in HDL (mg/dL)  G1: 2.43 (6.20)  G2: 0.48 (5.15)  P= 0.20  Change in triglycerides (mg/dL)  G1: -8.71 (36.29)  G2: -36.34 (243.30)  P= 0.55 |
| Delahanty et al.^45^ | US | 211 | 55% | Randomized controlled trial (RCT) | Adults (age > 18 years, mean age = 62 years), BMI > 25 kg/m2 or 23 kg/m2 if asian ancestry (mean BMI = 35 kg/m2), HbA1c 65-11.5%, Systolic blood pressure (SBP) < 160 mmHg, diastolic blood pressure (DBP) < 100 mmHg, 31 % taking insulin, with type 2 diabetes  G1 (Individual medical nutrition therapy - MNT, n=69) G2 (In-person group lifestyle intervention, n=70) G3 (Telephone group lifestyle intervention, n=72) | G1 (MNT): - Frequency: not contain any prespecified approach, content, or number of sessions - Setting: individual - Staff: registered dietitian   G2 (In-person lifestyle intervention): - Frequency: 19 group sessions (60–90min) in the first 6 months (14 weekly sessions followed by 5 bi-weekly) and 18 monthly sessions from 6 months until the end of year 2 + 5 individual sessions over the two-year  - Setting: groups (4–12 patients) + individual - Staff: registered dietitians   G3 (Telephone lifestyle intervention): - Frequency: 19 group sessions (60–90min) in the first 6 months (14 weekly sessions followed by 5 bi-weekly) and 18 monthly sessions from 6 months until the end of year 2 + 5 individual sessions over the two-year  - Setting: groups (4–12 patients) + individual - Staff: registered dietitians | Baseline, 6 months, 12 months, 18 months, 24 months (intervention completion), and 36 months | Mean weight loss at 6 months  G1: 2,6%  G2 and G3: 6,7% | Primary | Weight, BMI |
| Delahanty et al.^46^ | US | 211 | 55% | Randomized controlled trial (RCT) | Adults (age > 18 years, mean age = 62 years), BMI > 25 kg/m2 or 23 kg/m2 if asian ancestry (mean BMI = 35 kg/m2), HbA1c 65-11.5%, Systolic blood pressure (SBP) < 160 mmHg, diastolic blood pressure (DBP) < 100 mmHg, 33% taking insulin, with type 2 diabetes  G1 (Individual medical nutrition therapy - MNT, n=69) G2 (In-person group lifestyle intervention, n=70) G3 (Telephone group lifestyle intervention, n=72) | G1 (MNT): - Frequency: not contain any prespecified approach, content, or number of sessions - Setting: individual - Staff: registered dietitian   G2 (In-person lifestyle intervention): - Frequency: 19 group sessions (60–90min) in the first 6 months (14 weekly sessions followed by 5 bi-weekly) and 18 monthly sessions from 6 months until the end of year 2 + 5 individual sessions over the two-year  - Setting: groups (4–12 patients) + individual - Staff: registered dietitians   G3 (Telephone lifestyle intervention): - Frequency: 19 group sessions (60–90min) in the first 6 months (14 weekly sessions followed by 5 bi-weekly) and 18 monthly sessions from 6 months until the end of year 2 + 5 individual sessions over the two-year  - Setting: groups (4–12 patients) + individual - Staff: registered dietitians | Baseline, 6 months, 12 months, 18 months, 24 months (intervention completion), and 36 months | Weight loss from baseline  6 months G1: -1.1% (0.2–2.0%)  G2: -5.6% (4.4–6.8%)  G3: -4.6% (3.3–6.0%)   12 months G1: -2.0% (0.9–3.0%) G2: -4.6% (3.1–6.1%) G3: -4.8% (3.3–6.2%)  At least 5% weight loss at 12 months  G1: 22%  G2: 49%  G3: 44% G2 vs. G1: P< 0.001 G3 vs. G1: P< 0.001 G2 vs. G3: P=0.63 | Primary | 6 months:  HbA1c change from baseline G1: -0.3 (1.1 SD) G2: -0.6 (0.8 SD) G3: -0.5 (1.2 SD)  Systolic blood pressure change (mmHg) G1: 0.0 (14.7 SD) G2: -4.0 (15.0 SD) G3: -2.3 (14.4 SD)  Diastolic blood pressure change (mmHg)  G1: -0.9 (7.6 SD) G2: -1.3 (8.1 SD) G3: -2.0 (8.2 SD)  Total cholesterol change (mg/dl)  G1: 0.0 (25.1 SD) G2: -6.2 (27.0 SD) G3: -4.3 (27.9 SD)  High-density lipoprotein change (mg/dl)  G1: 1.0 (6.0 SD) G2: 1.7 (7.1 SD) G3: 2.9 (6.8 SD)  Low-density lipoprotein change (mg/dl) G1: -3.3 (22.4 SD) G2: -3.0 (21.0 SD) G3: -3.5 (26.0 SD)  Triglyceride change (mg/dl) G1: 12.5 (79.7 SD) G2: -56.0 (252.6) G3: -21.3 (80.7)  12 months:  HbA1c change from baseline G1: -0.4 (1.2 SD) G2: -0.4 (0.8 SD) G3: -0.2 (1.3 SD) G2 vs. G1: P= 0.39 G3 vs. G1: P= 0.98 G2 vs. G3: P= 0.43  Systolic blood pressure change (mmHg) G1: 1.8 (15.8 SD) G2: -2.0 (14.9 SD) G3: -1.0 (14.7 SD) G2 vs. G1: P= 0.09 G3 vs. G1: P= 0.28 G2 vs. G3: P= 0.54  Diastolic blood pressure change (mmHg) G1: 1.5 (8.0 SD) G2: -0.3 (7.4 SD) G3: -1.5 (7.6 SD) G2 vs. G1: P= 0.42 G3 vs. G1: P= 0.13 G2 vs. G3: P= 0.49  Total cholesterol change (mg/dl) G1: -1.6 (30.4 SD) G2: -7.6 (29.4 SD) G3: 1.1 (28.9 SD) G2 vs. G1: P= 0.14 G3 vs. G1: P= 0.82 G2 vs. G3: P= 0.20  High-density lipoprotein change (mg/dl) G1: 1.8 (5.7 SD) G2: 3.7 (6.4 SD) G3: 3.8 (6.5) G2 vs. G1: P= 0.21 G3 vs. G1: P= 0.059 G2 vs. G3: P= 0.53  Low-density lipoprotein change (mg/dl) G1: -3.3 (23.5 SD) G2: -7.7 (24.4 SD) G3: -0.6 (24.7 SD) G2 vs. G1: P= 0.57 G3 vs. G1: P= 0.78 G2 vs. G3: P= 0.39  Triglyceride change (mg/dl) G1: 5.3 (72.9 SD) G2: -59.0 (265.4 SD) G3: -6.5 (107.0 SD) G2 vs. G1: P= 0.019 G3 vs. G1: P= 0.40 G2 vs. G3: P= 0.12 |
| Foster et al.^43^ | US | 100 | 59 | Randomized controlled trial (RCT) | Adults (age range = 21-75 years, mean age = 55.6±10.6 years), weight = 102.9±18.4 kg, BMI = 35.8±5.3 kg/m2, HbA_1c_ 7.7±1.3%, 59 African Americans, 36 Caucasians and 3 Asian Americans, with type 2 diabetes  G1 (Lifestyle intervention, Portion-controlled Diet - PCD, n=50)  G2 (Diabetes Self-Management Education - DSME, n=50) | G1 (Lifestyle intervention, PCD): - Frequency: 9 group sessions (90 min), monitoring blood glucose at least twice daily with a glucometer and test strips. Meeting with a study physician at week 16 to review any changes in health that were observed at the 3-month study assessment.  - Setting: group (8–12 persons) - Staff: lifestyle interventionists  G2 (DSME): - Frequency: 9 group sessions (90 min), monitoring blood glucose at least twice daily with a glucometer and test strips. Meeting with a study physician at week 16 to review any changes in health that were observed at the 3-month study assessment.  - Setting: group (8–12 persons) - Staff: certified diabetes educators | Baseline and months 6 | Adjusted changes from baseline to 6-month  Weight (kg)  G1: -7.3 (-8.8 to  -5.8)  G2: -2.2 (-3.7 to  -0.7)  P < 0.0001  Body mass index (kg m^-2^  G1: -2.5 (-3.0 to  -2.0)  G2: -0.7 (-1.2 to  -0.2)  P < 0.0001 | Primary | Adjusted changes from baseline to 6 months  Waist circumference (cm)  G1: -6.5 (-7.8 to -5.3)  G2: -2.9 (-4.1 to -1.7)  P < 0.0001  Hemoglobin A_1C_ (%)  G1: -0.7 (-1.0 to -0.4)  G2: -0.4 (-0.7 to -0.1)  P = 0.021  Fasting glucose (mg dl^-1^)  G1: -16.5 (-33.0 to +0.1)  G2: -12.8 (-29.3 to +3.7)  P = 0.217^a^  Systolic blood pressure (mm Hg)  G1: -6.6 (-11.0 to -2.2)  G2: -1.9 (-6.2 to +2.5)  P = 0.044^a^  Diastolic blood pressure (mm Hg)  G1: -2.2 (-4.6 to +3.0)  G2: -1.2 (-3.6 to +1.2)  P = 0.359  Triglycerides (mg dl^-1^)  G1: -26.3 (-42.4 to -10.3)  G2: -20.3 ( -36.3 to -4.3)  P = 0.343^a^  Total cholesterol (mg dl^-1^)  G1: -8.6 (-15.9 to -1.2)  G2: -7.8 (-15.1 to -0.5)  P = 0.808  High-density lipoprotein (mg dl^-1^)  G1: -0.1 (-1.9 to +1.7)  G2: -1.6 (-3.4 to +0.2)  P = 0.113  Low-density lipoprotein (mg dl^-1^)  G1: -4.3 (-11.0 to +2.4)  G2: -0.8 (-7.6 to +5.9)  P = 0.320  hs-CRP (mg l^-1^)  G1: -1.0 (2.5 to þ 0.5)  G2: - 1.2 (2.6 to 0.3)  P = 0.883  ^a^ Indicates that P-values were obtained from a linear mixed-effects model on the log-transformed outcome, which were similar in direction and significance to analyses performed on the raw outcome.  ^b^ Data were obtained for only 49 of 50 participants |
| Gamiochipi et al.^47^ | Mexico | 199 | 78% | Randomized controlled trial (RCT) | Adults (mean age = 49.5 years), weight 73.5 kg (35% of the patients were overweight and 59% were obese; the remaining 6% were within normal weight limits but exhibited A1c > 7%; regarding A1c, 84% of patients had values of A1c >= 7%; the remaining 26 (16%) were all either overweight or obese), not taking insulin, with type 2 diabetes  G1 (IIEV - intensive lifestyle intervention, n=104)  G2 (COED - collaborative education intervention, n=95) | G1 (IIEV): - Frequency: 16 weekly sessions over the course of the 6 months + telephone calls, psychologist consultations, house calls  - Setting: individual - Staff: certified nutritionists, psychologist  G2 (COED): - Frequency: 16 weekly sessions over the course of the 6 months - Setting: individual - Staff: diabetes educators | Baseline, 3 and 6 months | Weight loss (kg) G1: 2.18 G2: 0.85  At least 5% weight loss at 6 months  G1: 26,1%  G2: 13,6% | Primary | Diastolic BP (mmHg) G1: -3.87  G2: - 1.14  (p = 0.042)  Systolic BP (mmHg) G1: -5.19 G2: - 1.40  (p = 0.497)  Total Cholesterol (mg/dl) G1: 2.49 G2: 7.08 (p = 0.890)  HDL (mg/dl) G1: 8.20 G2: 3.30 (p = 0.9167)  LDL (mg/dl) G1: 9.35 G2: 12.06 (p = 0.858)  Triglycerides (mg/dl) G1: 56.67 G2: 17.07 (p = 0.365) |
| Hankonen et al.^41^ | UK | 478 | 38 | Randomized controlled trial (RCT) | Adults (age range: 40–69 years, mean age = 60 years), with type 2 diabetes  G1 (ADDITION-Plus, n=239)  G2 (Control group, n=239) | G1 (ADDITION-Plus ):  intensive diabetes treatment + facilitator-led, theory-based, individual-level behavior change intervention - Frequency: 1-h introductory meeting followed by 6 30-min meetings and 4 brief phone calls, during a year  - Setting: individual - Staff: practice nurse, trained lifestyle facilitators  G2 (COED):  intensive diabetes treatment - Frequency: none - Setting: individual  - Staff: practice nurse | Baseline and 1 year | BMI baseline G1: 32.7 G2: 32.8  BMI at 1 year  G1: 32.1  G2: 32.3 | Primary | Baseline:  Diastolic BP (mmHg) G1: 81.6 G2: 79.1  (95%CI: -0.07 – 2.92)  Systolic BP (mmHg) G1: 138.2 G2: 13.4  (95%CI: -0.92 – 4.43)  Total Cholesterol (mg/dl) G1: 4.96 G2: 4.90 (95%CI: -0.16 – 0.15)  HDL (mg/dl) G1: 1.17 G2: 1.20 (95%CI: -0.02 – 0.05)  LDL (mg/dl) G1: 2.89 G2: 2.87 (95%CI: -0.19 – 0.08)  1 year:  Diastolic BP (mmHg) G1: 77.7 G2: 75.1  (95%CI: -0.07 – 2.92)  Systolic BP (mmHg) G1: 133.1 G2: 128.3  (95%CI: -0.92 – 4.43)  Total Cholesterol (mg/dl) G1: 4.33 G2: 4.31 (95%CI: -0.16 – 0.15)  HDL (mg/dl) G1: 1.19 G2: 1.19 (95%CI: -0.02 – 0.05)  LDL (mg/dl) G1: 2.29 G2: 2.34 (95%CI: -0.19 – 0.08) |
| Kuna et al^40^ | US | 264 | 59% | Randomized controlled trial (RCT) | Adults (age range: 45-76 years, mean age = 61.3 ± 6.5 years), BMI ≥ 25 kg/m2 (or ≥ 27 kg/m2 if taking insulin), mean BMI = 36.6 ± 5.7, HbA1C < 11%, systolic pressure < 160 mm Hg, diastolic pressure < 100 mm Hg, with type 2 diabetes and obstructive sleep apnea (OSA)  G1 (Intensive Lifestyle Intervention - ILI, n=125)  G2 (Diabetes Support and Education - DSE, n=139) | G1 (ILI): Phase I: Months 1 to 6 - Frequency: Weekly - Setting: 3 group (from 10 to 20 persons, of 60 to 75 minutes), one individual (20 to 30 minutes) - Staff: lifestyle counselor  Months 7 to 12 - Frequency: 3 per month - Setting: 2 group, 1 individual - Staff: lifestyle counselor, study physician (or nurse practitioner)  Phase II: Years 2 to 4 - Frequency: Minimum of 1 per month - Setting: 1 individual with minimum of 1 additional contact by phone, mail, or e-mail - Staff: lifestyle counselor  General staff: registered dietitian, behavioral psychologist (or other mental health professional) and an exercise specialist supported by a program coordinator, a physician, and a diabetes educator (often a nurse)  G2 (DSE): - Frequency: 3 group session annually - Setting: group - Staff: certified diabetic educator and a nutritionist + usual medical care, provided by their own primary care physicians | Baseline, 1, 2 and 4 years | Change in weight (kg) from baseline to 1 year  G1: -10.7 ± 0.7  p < 0.001  G2: -0.5 ± 0.7  p = 0.50  2 years  G1: -7.4 ± 0.7  p < 0.001  G2: -0.8 ± 0.7  p = 0.24  4 years  G1: -5.2 ± 0.7  p < 0.001  G2: -0.8 ± 0.7  p = 0.22 | Primary | Changes from baseline to 1 year  AHI (events/h)  G1: -5.7 ± 1.5  p < 0.001  G2: 4.0 ± 1.4  p= 0.004  Neck circumference (cm)  G1: -1.16 ± 0.26  p < 0.001  G2: 0.12 ± 0.25  p= 0.62  Waist circumference (cm)  G1: -9.09 ± 0.69  p < 0.001  G2: -0.35 ± 0.66  p= 0.59  2 years  AHI (events/h)  G1: -3.8 ± 1.5  p= 0.011  G2: 4.2 ± 1.4  p= 0.003  Neck circumference (cm)  G1: -1.15 ± 0.27  p < 0.001  G2: -0.21 ± 0.26  p= 0.42  Waist circumference (cm)  G1: -5.99 ± 0.69  p < 0.001  G2: -0.71 ± 0.67  p= 0.29  4 years  AHI (events/h)  G1: -4.0 ± 1.6  p= 0.015  G2: 3.7 ± 1.6  p= 0.02  Neck circumference (cm)  G1: -1.10 ± 0.30  p < 0.001  G2: -0.57 ± 0.29  p= 0.05  Waist circumference (cm)  G1: -3.42 ± 0.71  p < 0.001  G2: 0.17 ± 0.68  p= 0.79 |
| Lutes et al.^38^ | US | 200 | 100% | Randomized controlled trial (RCT) | Adults (age range: 19-75 years, mean age = 53.45 ± 10.24 years) African American women, study population was characterized as rural and impoverished, mean BMI = 37.67 ± 8.02 kg/m2, HbA1c ≥ 7.0% (mean 9.09 ± 1.83), 60% using insulin, 67% using food assistance program, with class II obesity and uncontrolled type 2 diabetes  G1 (Small Changes Intervention group, n=100)  G2 (Mail-based education group, n=100) | G1 (Small Changes Intervention group): - Frequency: 16 phone sessions (20 -30 min) across 12 months - Setting: individual - Staff: primary care providers (CHW)  G2 (Mail-based education group): - Frequency: 16 educational mailings across 12 months - Setting: individual - Staff: Academy of Nutrition and Dietetics | Baseline, 6 and 12 months | Weight (kg) at baseline  G1: 98.09 ± 21.21  G2: 104.20 ± 25.36  Weight (kg) at 6 months  G1: 97.72 ± 21.08  G2: 104.42 ± 25.35  Weight (kg) at 12 months  G1: 96.74 ± 22.13  G2: 103.81 ± 25.74  95% CI  G1: 92.03-101.06  G2: 98.49-107.71  Weight total change:  G1: -1.35 ± 6.22  G2: -0.39 ± 4.57 | Primary | HbA1c at baseline  G1: 9.13 ± 1.79  G2: 9.05 ± 1.88  HbA1c at 6 months  G1: 8.87 ± 1.92  G2: 8.89 ± 2.11  HbA1c at 12 months  G1: 8.84 ± 1.98  G2: 9.10 ± 2.24  95% CI  G1: 8.61-9.28  G2: 8.67-9.36  HbA1c total change:  G1: -0.29 ± 1.84  G2: +0.05 ± 1.61  Systolic BP at baseline  G1: 134.71 ± 22.01  G2: 137.75 ± 20.02  Systolic BP at 6 months  G1: 138.16 ± 19.43  G2: 145.22 ± 22.14  Systolic BP at 12 months  G1: 134.93 ± 22.25  G2: 136.73 ± 21.10  95% CI  G1: 132.65-139.22  G2: 136.49-143.31  Systolic BP total change:  G1: +0.22 ± 25.33  G2: -1.01 ± 20.46  Diastolic BP at baseline  G1: 85.41 ± 13.03  G2: 84.74 ± 11.83  Diastolic BP at 6 months  G1: 85.73 ± 12.23  G2: 88.50 ± 12.02  Diastolic BP at 12 months  G1: 82.54 ± 13.52  G2: 85.40 ± 10.84  95% CI  G1: 82.71-86.41  G2: 84.29-88.13  Diastolic BP total change:  G1: -2.87 ± 1.52  G2: +0.66 ± 13.24 |
| Moncrieft et al.^39^ | US | 111 | 71.2 | Randomized controlled trial (RCT) | Adults (age range: 18-70 years, mean age = 54.81 ± 7.36 years), BMI ≥ 27 kg/m2 (mean = 32.60 ± 4.70 kg/m2), minority status (84.7% Hispanic, 10.8% Black) and low-income range, 16.22% taking Antidepressive medicines, 83.8% taking Antihyperglycemic medicines, with moderate depressive symptoms (BDI-II total score ≥ 11) and type 2 diabetes  G1 (Community Approach to Lifestyle Modification for Diabetes - CALM-D, n=57)  G2 (Usual Care, n=54) | G1 (CALM-D): - Frequency: 17-session (1.5 to 2 hours) of which the first 2 individual sessions followed by 2 weekly and 4 bi-weekly group sessions. The remaining 9 group sessions were scheduled monthly.  - Setting: individual, group - Staff: trained therapists  G2 (Usual Care): None | Baseline, 6 and 12 months | Weight (kg) at baseline  G1: 85.04 ± 12.22  G2: 85.57 ± 16.20  Weight (kg) at 6 months  G1: 81.78 ± 12.56  G2: 85.21 ± 16.05  Weight (kg) at 12 months  G1: 82.03 ± 12.58  G2: 84.19 ± 15.48 | Primary | HbA1c (%) at baseline  G1: 7.67 ± 1.40  G2: 7.77 ± 1.23  HbA1c (%) at 6 months  G1: 7.36 ± 1.48  G2: 7.89 ± 1.45  HbA1c (%) at 12 months  G1: 7.39 ± 1.46  G2: 7.93 ± 1.37  BDI-II total at baseline  G1: 19.28 ± 7.08  G2: 21.21 ± 7.12  BDI-II total at 6 months  G1: 10.75 ± 7.76  G2: 16.09 ± 9.15  BDI-II total at 12 months  G1: 9.85 ± 8.86  G2: 16.00 ± 10.80  eGFR (ml/min) at baseline  G1: 88.57 ± 15.35  G2: 85.17 ± 20.47  eGFR (ml/min) at 6 months  G1: 91.90 ± 20.67  G2: 91.33 ± 18.91  eGFR (ml/min) at 12 months  G1: 91.15 ± 14.61  G2: 85.61 ± 17.30 |
| Osama and Shehab^58^ | Saudi Arabia | 100 | 44% | Randomized controlled trial (RCT) | Adults (age range: 35-45 years, mean age = 37 years), BMI range: 32-36 kg/m2 (mean BMI = 33.01 kg/m2), free from other comorbidities, with type 2 diabetes  G1 (Group A, n=50) G2 (Group B, n=50) | G1 (Group A): - Frequency: 3 sessions/week for 3 months of 40 minutes moderate intensity aerobic exercise + weekly nutritional counseling - Setting: individual - Staff: dietitian, physician   G2 (Group B):  None | Baseline and after 3 months | BMI change (kg/m2) G1: from 32.86 ± 5.29 to 30.13 ± 4.32 (P <0.05) G2: from 33.15 ± 4.87 to 33.45 ± 4.16 (P> 0.05) | Primary | Change from baseline Total cholesterol (mg/dl) G1: 192.30 ± 12.86 --> 176.54 ± 11.66 (P <0.05) G2: 193.54 ± 11.22 --> 195.12 ± 10.25 (P>0.05)  HDL-c (mg/dl) G1: 34.54 ± 2.71 --> 36.35 ± 2.48 (P <0.05) G2: 33.73 ± 2.95 --> 32.81 ± 2.74 (P>0.05)  LDL-c (mg/dl) G1: 132.93 ± 9.78 --> 120.27 ± 8.94 (P <0.05) G2: 133.64 ± 9.03--> 133.88 ±8.72 (P>0.05)  Triglyceride (mg/dl) G1: 154.15 ± 10.21 --> 129.61 ± 9.83 (P <0.05) G2: 155.18 ± 9.82 --> 156.11 ± 9.23 (P>0.05) |
| St Jules et al.^37^ | UK | 256 | 50% | 2 X 2 Factorial design Randomized controlled trial (RCT) | Adults (age ≥ 40, mean age = 65 (9) years), BMI ≥ 27 kg/m2 (mean BMI = 33.8 (5.1) kg/m2), with type 2 diabetes and chronic kidney disease  G1 (Control group - ADVICE, n=64)  G2 (Social Cognitive Theory–based Behavioral Group Counseling - SCT, n=64)  G3 (Technology-based self-monitoring MONITORING, n=64)  G4 (COMBINED, n=64) | G1 (ADVICE):  - Frequency: Written information on the objectives was initially provided by mail.  During the next intervention period (6 months), monthly sending of 1-page educational handouts - Setting: individual - Staff: dietitian  G2 (SCT):  - Frequency: 14 WebEx video-conferencing sessions (1 hour). Weekly frequency for the first 4  weeks, then every other week up to 20 weeks - Setting: group (12-15 individuals) - Staff: dietitian  G3 (MONITORING):  - Frequency: 14 WebEx video-conferencing sessions (1 hour). Weekly frequency for the first 4  weeks, then every other week up to 20 weeks. Daily compilation of MyNetDiary. - Setting: group (12-15 individuals), individual - Staff: dietitian  G4 (COMBINED):  - Frequency: 14 WebEx video-conferencing sessions (1 hour). Weekly frequency for the first 4  weeks, then every other week up to 20 weeks. Daily compilation of MyNetDiary. - Setting: group (12-15 individuals), individual - Staff: dietitian | Baseline, 3 and 6 months | Weight loss, >5% [n (%)] at 3 months  G1: 5 (12%)  G2: 3 (7%)  G3: 10 (20%)  G4: 12 (24%)  P = 0.08  Weight loss, >5% [n (%)] at 6 months  G1: 7 (18%)  G2: 7 (14%)  G3: 9 (20%)  G4: 14 (27%)  P = 0.39  Weight change (kg) at 3 months G1: -1.7 (3.6) G2: -1.4 (2.6)  G3: -2.7 (2.7)  G4: -2.2 (3.6) P = 0.11  Weight change (kg) at 6 months G1: -1.2 (4.3) G2: -1.4 (3.1)  G3: -2.3 (3.4)  G4: -2.7 (4.4)  P= 0.19 | Primary | Changes from baseline to 3 months:  Urine Na:Cr (mg/g) G1: -161 (1,007) G2: 15 (1,959)  G3: -180 (1,552)  G4: 17 (1,222) P = 0.91  Urine P:Cr (mg/g)  G1: 10 (192) G2: 39 (341)  G3: -37 (141)  G4: 22 (194)  P = 0.55  HbA1c (%)  G1: -0.3 (1.1) G2: -0.1 (0.9)  G3: -0.4 (0.9)  G4: -0.4 (0.9)  P = 0.41  SBP (mm Hg)  G1: -4 (20) G2: -1 (16)  G3: 4 (17)  G4: 0 (18) P = 0.25  DBP (mm Hg)  G1: -4 (14) G2: 1 (11)  G3: 3 (11)  G4: 0.0 (11) P = 0.09  cf-PWV (m/s)  G1: 0.6 (2.0)  G2: -0.3 (2.6)  G3: 0.8 (3.3)  G4: -0.4 (2.4) P = 0.18  Chol (mg/dL)  G1: -4 (20)  G2: -4 (34)  G3: 2 (22)  G4: -9 (28)  P = 0.24  HDL (mg/dL)  G1: 0 (7) G2: 1 (6)  G3: 1 (6)  G4: 1 (6)  P = 0.91  LDL (mg/dL)  G1: -2 (17)  G2: -3 (28)  G3: 2 (19)  G4: -8 (24)  P = 0.18  Trig (mg/dL)  G1: -8 (69)  G2: -8 (61)  G3: -2 (62)  G4: -8.6 (75)  P = 0.97  6 months:  Urine Na:Cr (mg/g)  G1: -137 (1,262) G2: 87 (1,346)  G3: -100 (1,334)  G4: 98 (1,107)  P = 0.81  Urine P:Cr (mg/g)  G1: 28 (240) G2: 31 (184)  G3: 7 (205)  G4: -15 (201)  P = 0.77  HbA1c (%)  G1: -0.3 (1.1) G2: -0.1 (1.0)  G3: -0.3 (0.9)  G4: -0.3 (0.9) P = 0.64  SBP (mm Hg)  G1: -4 (24) G2: -3 (16)  G3: -2 (15)  G4: -4 (19) P = 0.92  DBP (mm Hg)  G1: -4 (15)  G2: -1 (10)  G3: 0 (11)  G4: -2.3 (12) P = 0.47  cf-PWV (m/s)  G1: -0.3 (2.4) G2: 0.2 (2.2)  G3: 0.7 (2.4)  G4: -0.6 (2.2) P = 0.10  Chol (mg/dL)  G1: -4 (19) G2: -7 (28)  G3: 1 (23)  G4: -2 (35)  P = 0.55  HDL (mg/dL)  G1: 1 (6) G2: 2 (8)  G3: 1 (7)  G4: 1 (9)  P = 0.95  LDL (mg/dL)  G1: -1 (15)  G2: -4 (29)  G3: -1 (22)  G4: -4 (29)  P = 0.89  Trig (mg/dL)  G1: -23 (79) G2: -16 (73)  G3: 5 (64)  G4: -3.9 (88)  P = 0.35 |
| The Look AHEAD Research Group^42,48-56^ | US | 5145 | 59.5% | Multicenter (16 centres), randomized controlled trial (RCT) | Adults (age range: 45-76 years; mean age: 59 years), overweight and obese (BMI ≥25kg/m2 or ≥27kg/m2 if taking insulin; mean BMI: 36 kg/m2), with type 2 diabetes (determined by self-report with verification), 15.4% taking insulin, 84% of participants have hypertension, 94% have metabolic syndrome and 14% have a history of cardiovascular disease | G1 (DSE): - Frequency: 3 group educational/social support per year for the first 4 years - Setting: 3 group - Staff: certified diabetic educator and a nutritionist + usual medical care, provided by their own primary care physicians  G2 (ILI): Phase I: Months 1 to 6 - Frequency: Weekly - Setting: 3 group (from 10 to 20 persons, of 60 to 75 minutes), one individual (20 to 30 minutes) - Staff: lifestyle counselor  Months 7 to 12 - Frequency: 3 per month - Setting: 2 group, 1 individual - Staff: lifestyle counselor, study physician (or nurse practitioner)  Phase II: Years 2 to 4 - Frequency: Minimum of 1 per month - Setting: 1 individual with minimum of 1 additional contact by phone, mail, or e-mail - Staff: lifestyle counselor  Phase III: Year 5+ - Frequency: Monthly recommended - Setting: Individual - Staff: lifestyle counselor  General staff: registered dietitian, behavioral psychologist (or other mental health professional) and an exercise specialist supported by a program coordinator, a physician, and a diabetes educator (often a nurse) | Baseline, year 1, year 2, year 3, year 4, and extended follow-up every other year (they wanted to do assessments over 13.5 years but the study was early stopped when the median follow-up was 9.6 years.) | Weight loss at year 1 ILI: 8.5% ± 0.2 of initial weight DSE: 0.6% ± 0.2 of initial weight (P <0.001)  Weight loss at Year 4 ILI: 4.4% ± 0.2 of initial weight DSE: 0.7% ± 0.2 of initial weight  (P < 0.001)  Weight loss at Year 8 ILI: 4.7% ± 0.2 of initial weight DSE: 2.1% ± 0.2 of initial weight  (P < 0.001) (Mean losses, including participants who had bariatric surgery, were 5.3% ± 0.2% and 2.7% ± 0.2%, respectively)   Weight loss from baseline at the end of the study ILI: 6.0% DSE: 3.5% | Secondary | Changes at year 1 from baseline  Fasting glucose (mg/dl) ILI: -21.5 ± 0.9 DSE: -7.2 ± 0.9 (P < 0.001)  Glycated hemoglobin (%) ILI: -0.64 ± 0.02 DSE: -0.14 ± 0.02 (P < 0.001)  Systolic blood pressure (mmHg) ILI: -6.8 ± 0.4 DSE: -2.8 ± 0.3 (P < 0.001)  Diastolic blood pressure (mmHg) ILI: -3.0 ± 0.2 DSE: -1.8 ± 0.2 (P < 0.001)  LDL cholesterol (mg/dl) ILI: -5.2 ± 0.6 DSE: -5.7 ± 0.6 (P = 0.49)  HDL cholesterol (mg/dl) ILI: 3.4 ± 0.2 DSE: 1.4 ± 0.1 (P < 0.001)  Triglycerides (mg/dl) ILI: -30.3 ± 2.0 DSE: -14.6 ± 1.8 (P < 0.001)  Changes at year 4 from baseline  Glycated hemoglobin (%) ILI: -0.36 DSE: -0.09 (P < 0.0001)  Systolic blood pressure (mmHg) ILI: −5.33 DSE: −2.97 (P < 0.0001)  Diastolic blood pressure (mmHg) ILI: −2.92 DSE: −2.48 (P = 0.012)  LDL cholesterol (mg/dl) ILI: −11.27 DSE: −12.84 (P = 0.009)  HDL cholesterol (mg/dl) ILI: 3.67 DSE: 1.97 (P < 0.0001)  Triglycerides (mg/dl) ILI: −25.56 DSE: −19.75 (P = 0.0006) |
| Wang et al.^29^ | UK | 26 | 58% | Pilot randomized controlled trial (RCT) | Adults (age range: 21-75 years, mean age = 56.4 years), BMI > 25 kg/m2 (BMI range: 27.4-51.1, mean BMI = 38.1 kg/m2), 69% African Americans with type 2 diabetes  G1 (Paper group, n=9)  G2 (Mobile group, n=11)  G3 (Usual care and education – Control group, n=6) | G1 (Mobile group): - Frequency: 11 group sessions weekly for month 1, biweekly for months 2 and 3, and monthly for months 4 to 6 and an individual session after month 3. The participants used the LoseIt! smartphone app for self-monitoring of diet, PA, and weight and the Diabetes Connect app connected with MyGlucoHealth, a Bluetooth-enabled glucometer - Setting: individual, group - Staff: primary care physicians, diabetes educators, lifestyle counsellors  G2 (Paper group): - Frequency: 11 group sessions weekly for month 1, biweekly for months 2 and 3, and monthly for months 4 to 6 and an individual session after month 3 - Setting: individual, group - Staff: primary care physicians, diabetes educators, lifestyle counsellors  G3 (Usual care and education – Control group): - Frequency: individual visits or a series of two interactive group classes - Setting: individual, group - Staff: primary care physicians, diabetes educators | Baseline, 3 and 6 months | Weight (kg), median (Q1, Q3) at baseline  G1: 233.6 (179.8, 295.4)  G2: 243.6 (222.2, 321.8)  G3: 201.2 (195.8, 213.8)  P=0.48^a^  % weight change at 3 months, median (Q1, Q3)  G1: 0.5 (−2.9, 2.2)  G2: −1.0 (−1.6, −0.1)  G3: 2.1 (0.1, 4.2)  P=0.16^a^  % weight change at 3 months, median (Q1, Q3)  G1: −1.8 (−4.2, −0.3)  G2: 0.4 (−2.3, 1.5)  G3: 1.6 (−4.1, 3.8)  P=0.16^a^  Q1: 25th percentile Q3: 75th percentile  ^a^ P values obtained from Kruskal-Wallis test | Secondary | HbA1c at baseline  G1: 8.4 (2.3)  G2: 10.4 (2.4)  G3:8.9 (2.4)  P = 0.20  HbA1c at 3 months  G1: 7.3 (1.1)  G2: 8.5 (1.4)  G3: 8.5 (1.7)  P = 0.13  HbA1c at 6 months  G1: 6.9 (1.0)  G2: 9.1 (1.8)  G3: 8.9 (1.6)  P = 0.01  P values obtained from analysis of variance |
| West et al.^48^ | US | 2570 | 58% | Multicenter (16 centres), randomized controlled trial (RCT) | Adults (mean age: 58.6 ± 0.1 years), overweight and obese (BMI ≥ 25 kg/m2 or ≥ 27 kg/m2 if taking insulin; 15,7% with BMI < 30 kg/m2, 35,7% with BMI =-34.99 kg/m2, 48,6% with BMI ≥ 35 kg/m2), with type 2 diabetes, 16% self-identified as African American, 13% as Hispanic, 63% as non-Hispanic White and 8% as another minority group | G1 (ILI): Phase I: Months 1 to 6 - Frequency: Weekly - Setting: 3 group (from 10 to 20 persons, of 60 to 75 minutes), one individual (20 to 30 minutes) - Staff: lifestyle counselor  Months 7 to 12 - Frequency: 3 per month - Setting: 2 group, 1 individual - Staff: lifestyle counselor, study physician (or nurse practitioner)  Phase II: Years 2 to 4 - Frequency: Minimum of 1 per month - Setting: 1 individual with minimum of 1 additional contact by phone, mail, or e-mail - Staff: lifestyle counselor  Phase III: Year 5+ - Frequency: Monthly recommended - Setting: Individual - Staff: lifestyle counselor  General staff: registered dietitian, behavioral psychologist (or other mental health professional) and an exercise specialist supported by a program coordinator, a physician, and a diabetes educator (often a nurse) | Baseline, year 1, year 4 and year 8 | Weight change by racial/ethnic and sex subgroups within ILI  Weight (mean kg ± SE) at baseline  Non-Hispanic white  Men: 110 ± 0.7  Women: 96.9 ± 0.6  African American  Men: 112.9 ± 1.9  Women: 98.0 ± 1.0  Hispanic  Men: 100.4 ± 1.7  Women: 86.9 ± 1.1  Average (±SE) percent weight change from baseline to 1 year  Non-Hispanic white  Men: −10.0 ± 0.3  Women: −9.1 ± 0.3  African American  Men: −6.8 ± 0.5  Women: −6.8 ± 0.3  Hispanic  Men: −7.5 ± 0.6  Women: −8.1 ± 0.4  Average (±SE) percent weight change from baseline to 4 years  Non-Hispanic white  Men: −5.6 ± 0.3  Women: −4.5 ± 0.3  African American  Men: −3.2 ± 0.6  Women: −4.3 ± 0.5  Hispanic  Men: −4.1 ± 0.8  Women: −5.0 ± 0.6  Average (±SE) percent weight change from baseline to 8 years  Non-Hispanic white  Men: −5.1 ± 0.3  Women: −5.5 ± 0.4  African American  Men: −3.6 ± 0.9  Women: −6.3 ± 0.6  Hispanic  Men: −2.7 ± 1.2  Women: −5.8 ± 0.7  Average (±SE) weight change (kg) from baseline to 1 year  Non-Hispanic white  Men: −11.1 ± 0.3  Women: −8.9 ± 0.3  African American  Men: −7.7 ± 0.7  Women: −6.7 ± 0.3  Hispanic  Men: −7.4 ± 0.6  Women: −7.0 ± 0.4  Average (±SE) weight change (kg) from baseline to 4 years  Non-Hispanic white  Men: −6.4 ± 0.3  Women: −4.5 ± 0.3  African American  Men: −3.5 ± 0.7  Women: −4.4 ± 0.5  Hispanic  Men: −3.7 ± 0.9  Women: −4.5 ± 0.5  Average (±SE) weight change (kg) from baseline to 8 years  Non-Hispanic white  Men: −5.9 ± 0.4  Women: −5.6 ± 0.4  African American  Men: −3.7 ± 1.1  Women: −6.5 ± 0.6  Hispanic  Men: −2.3 ± 1.3  Women: −5.1 ± 0.6  Proportion of subgroup achieving ≥ 5% weight loss, n (%) at 1 year  Non-Hispanic white  Men: 559 (73.7)  Women: 574 (71.1)  African American  Men: 53 (55.8)  Women: 184 (62.4)  Hispanic  Men: 62 (66.7)  Women: 152 (67.9)  Proportion of subgroup achieving ≥ 5% weight loss, n (%) at 4 years  Non-Hispanic white  Men: 349 (49.1)  Women: 342 (44.5)  African American  Men: 38 (41.8)  Women: 116 (42.3)  Hispanic  Men: 31 (39.2)  Women: 102 (50.5)  Proportion of subgroup achieving ≥ 5% weight loss, n (%) at 8 years  Non-Hispanic white  Men: 321 (48.6)  Women: 386 (53.3)  African American  Men: 41 (47.7)  Women: 146 (56.4)  Hispanic  Men: 31 (41.9)  Women: 106 (54.4)  Proportion of subgroup achieving ≥ 10% weight loss, n (%) at 1 year  Non-Hispanic white  Men: 353 (46.6)  Women: 327 (40.5)  African American  Men: 25 (26.3)  Women: 71 (24.1)  Hispanic  Men: 25 (26.9)  Women: 95 (42.4)  Proportion of subgroup achieving ≥ 10% weight loss, n (%) at 4 years  Non-Hispanic white  Men: 190 (26.7)  Women: 172 (22.4)  African American  Men: 8 (8.8)  Women: 55 (20.1)  Hispanic  Men: 18 (22.8)  Women: 53 (26.2)  Proportion of subgroup achieving ≥ 10% weight loss, n (%) at 8 years  Non-Hispanic white  Men: 174 (26.4)  Women: 219 (30.2)  African American  Men: 17 (19.8)  Women: 82 (31.7)  Hispanic  Men: 19 (25.7)  Women: 61 (31.3) | Primary |  |

**Table S3.** Studies comparing behavioral interventions focusing on diet and exercise.

| **AUTHORS, YEAR** | **COUNTRY** | **N** | **%F** | **STUDY DESIGN** | **SAMPLE CHARACTERISTICS** | **CHARACTERISTICS OF THE INTERVENTION  (FREQUENCY, DURATION, SETTING, TYPE OF STAFF)** | **ASSESSMENT (AND FOLLOW-UP)** | **WEIGHT AND/OR BMI** | **WEIGHT OUTCOME** | **ANTHROPOMETRIC AND METABOLIC PARAMETERS** |
| --- | --- | --- | --- | --- | --- | --- | --- | --- | --- | --- |
| Carter et al.^31^ | Australia | 137 | 56 % | Randomized controlled trial (RCT) - Randomised noninferiority trial | Adults (age > 18 years, mean age = 61 years), BMI >= 27 kg/m2 (mean BMI = 36 kg/m2), 20% taking insulin, with type 2 diabetes  G1 (Intermittent, n = 70) G2 (Continuous, n = 67) | G1 (Intermittent): - Frequency: dietary counselling every 2 weeks for the first 3 months and every 2 to 3 months for the final 9 months - Setting: individual - Staff: dietitian  G2 (Continuous): - Frequency: dietary counselling every 2 weeks for the first 3 months and every 2 to 3 months for the final 9 months - Setting: individual - Staff: dietitian | Baseline, 3, 12 (end of intervention) and 24 months (follow-up) | Weight (kg) change at 24 months G2: -3.9 (1.1) [-6.0 to -1.7]* G1: -3.9 (1.1) [-6.1 to -1.7]*  BMI (kg/m2) change at 24 months G2: -1.4 (0.4) [-2.2 to -0.7]* G1: -1.3 (0.4) [-2.1 to -0.6]*   *Mean (SEM) [95% CI] | Secondary | HbA1c (%) change at 24 months G2: 0.4 (0.3) [-0.2 to 0.9] G1: 0.1 (0.2) [-0.3 to 0.5]  HbA1c (mmol/mol) change at 24 months G2: 4.4 () [-2.2 to 9.8] G1: 1.1 (2.2) [-3.3 to 5.5]  Fasting plasma glucose (mmol/L) change at 24 months G2: -0.3 (0.6) [-1.6 to 1.0] G1: -0.2 (0.5) [-1.2 to 0.8]  Total Cholesterol (mmol/L) change at 24 months G2: -0.3 (0.2) [-0.9 to 0.2] G1: 0.03 (0.2) [-0.3 to 0.4]  HDL-C (mmol/L) change at 24 months G2: -0.08 (0.06) [-0.2 to 0.04] G1: -0.1 (0.06) [-0.2 to 0.02]  LDL-C (mmol/L) change at 24 months G2: -0.2 (0.2) [-0.6 to 0.3] G1: 0.2 (0.2) [-0.2 to 0.5] |
| Goday et al.^36^ | Spain | 89 | 65% | Multicenter, randomized controlled trial (RCT) | Adults (age range: 30-65 years, mean years = 55 years), BMI range: 30-35 kg/m2 (mean BMI = 33.07 kg/m2), not taking insulin, with type 2 diabetes  G1 (VLCK diet group, n=45) G2 (LC diet group, n=44) | G1 (VLCK diet group):  - Frequency: nine individual sessions and a telephone contact every 15 days for 4 months - Setting: individual and group - Staff: specialist physician, expert dietician  G2 (LC diet group): - Frequency: nine individual sessions and a telephone contact every 15 days for 4 months - Setting: individual and group - Staff: specialist physician conducting the study, an assessment by an expert dietician + endocrinologist and a registered dietitian | Baseline, 4 months | BMI (kg/m2) at baseline G1: 33.3 (1.5 SD) G2: 32.9 (1.6 SD)  BMI (kg/m2) at 4 months G1: 27.9 (1.8 SD) G2: 31.0 (2.2 SD)  P value for change from baseline (G1): P < 0.0001 P value for change from baseline (G2): P < 0.0001  Body weight (kg) at baseline G1: 91.5 (11.4 SD) G2: 90.0 (11.3 SD)  Body weight (kg) at 4 months G1: 76.8 (9.1 SD) G2: 84.95 (13.6 SD)  P value for change from baseline (G1): P < 0.0001 P value for change from baseline (G2): P = 0.5960 | Primary | Waist (cm) at baseline G1: 108.1 (8.6 SD) G2: 105.8 (8.5 SD)  Waist (cm) at 4 months G1: 96.1 (7.6 SD) G2: 100.4 (9.2 SD)  P value for change from baseline (G1): P < 0.0001 P value for change from baseline (G2): P = 0.0481  Fasting glycemia (mg/dl) at baseline G1: 136.9 (34.4 SD) G2: 140.5 (43.1 SD)  Fasting glycemia (mg/dl) at 4 months G1: 108.9 (20.4 SD) G2: 123.3 (24.3 SD)  P value for change from baseline (G1): P < 0.0001 P value for change from baseline (G2): P = 0.1821  HbA1c (%) at baseline G1: 6.9 (1.1 SD) G2: 6.8 (1.0 SD)  HbA1c (%) at 4 months G1: 6.0 (0.7 SD) G2: 6.4 (0.8 SD)  P value for change from baseline (G1): P < 0.0001 P value for change from baseline (G2): P = 0.1453  Total cholesterol (mg/dl) at baseline G1: 200.1 (36.0 SD) G2: 199.4 (51.0 SD)  Total cholesterol (mg/dl) at 4 months G1: 187.5 (46.3 SD) G2: 191.7 (34.1 SD)  P value for change from baseline (G1): P = 0.1615 P value for change from baseline (G2): P = 0.4489  Triglycerides (mg/dl) at baseline G1: 150.5 (54.4 SD) G2: 176.1 (92.0 SD)  Triglycerides (mg/dl) at 4 months G1: 114.6 (57.2 SD) G2: 158.3 (61.0 SD)  P value for change from baseline (G1): P = 0.0040 P value for change from baseline (G2): P = 0.3308  LDL-c (mg/dl) at baseline G1: 112.7 (33.6 SD) G2: 109.8 (45.5 SD)  LDL-c (mg/dl) at 4 months G1: 110.6 (38.4 SD) G2: 107.1 (29.9 SD)  P value for change from baseline (G1): P = 0.7892 P value for change from baseline (G2): P = 0.7629  HDL-c (mg/dl) at baseline G1: 55.9 (11.1 SD) G2: 55.1 (11.7 SD)  HDL-c (mg/dl) at 4 months G1: 54.5 (11.3 SD) G2: 52.4 (10.0 SD)  P value for change from baseline (G1): P = 0.5728 P value for change from baseline (G2): P = 0.3017 |
| Otten et al.^33-34^ | Sweden | 32 | 34.5% | Randomized controlled trial (RCT) | Adults (age range: 30-70 years, mean age = 59 years), BMI 25–40 kg/m2 (mean BMI = 31.55 kg/m2), with type 2 diabetes  G1 (PD, n=16) G2 (PD-EX, n=16) | G1 (PD): - Frequency: 5 group sessions (the first 2 meetings were held during the first 2 weeks, and the following meetings took place once a month). The intervention lasts 12 weeks. - Setting: group - Staff: trained dietician    G2 (PD-EX): - Frequency: 5 group sessions (the first 2 meetings were held during the first 2 weeks, and the following meetings took place once a month) + 1-h sessions 3 times weekly. The intervention lasts 12 weeks. - Setting: group - Staff: trained dietician, personal trainers | Baseline and after 12 weeks | Weight (kg) change 0-12 weeks G1: −7.1 (−9.8, −5.6)*** G2: −7.0 (−9.7, −5.6)***  BMI (kg/m2) change 0-12 weeks G1: −2.4 (−3.1, −1.8)*** G2: −2.3 (−3.4, −2.2)***  ***P < 0.001 for the within-group change over time from baseline to 12 weeks  Both study groups showed a median body weight loss of 7 kg. Fat mass decreased by 5.7 kg in the G1 group and by 6.5 kg in the G2 group. | Primary | Waist circumference (cm) change 0-12 weeks G1: -9 (-12, -7)*** G2: -8 (-10, -7)***  HbA1c (%) change 0-12 weeks G1: −1.0 (−1.4, −0.5)** G2: −1.0 (−1.7, −0.6)**  HbA1c (mmol/mol) change 0-12 weeks G1: −11 (−15, −5)** G2: −11 (−18, −7)**  Fasting glucose (mmol/L) change 0-12 weeks G1: −0.9 (−1.8, −0.1)* G2: −2.0 (−3.0, −1.0)**  Fasting insulin (mIU/L) change 0-12 weeks G1: -8 (-16, -3)** G2: -4 (-8, -2)***  Systolic blood pressure (mmHg) change 0-12 weeks G1: -17 (-24, 0)** G2: -11 (-14, -7)***  Diastolic blood pressure (mmHg) change 0-12 weeks G1: -9 (-15, -6)*** G2: -10 (-13, -7)***  Total cholesterol (mmol/L) change 0-12 weeks G1: -0.3 (-0.6, 0.1) G2: -0.6 (-0.6, -0.4)**  Triglycerides (mmol/L) change 0-12 weeks G1: -0.6 (-1.5, -0.2)** G2: -0.5 (-1.0, -0.2)***  HDL (mmol/L) change 0-12 weeks G1: -0.01 (-0.08, 0.05) G2: 0.01 (-0.03, 0.07)  LDL (mmol/L) change 0-12 weeks G1: -0.1 (-0.4, 0.2) G2: -0.1 (-0.5, 0.1)  *P < 0.05 for the change over time from baseline to 12 weeks within the group **P < 0.01 for the change over time from baseline to 12 weeks within the group ***P < 0.001 for the change over time from baseline to 12 weeks within the group |
| Stomby et al.^57^ | Sweden | 28 | 36% | Randomized controlled trial (RCT) | Adults (age range: 30–70 years, mean age = 60 years), weight-stable (<5% weight change last 6 months), overweight or obese (BMI range: 25–40 kg/m2, mean BMI = 31.4 kg/m2), diagnosed with type 2 diabetes within 10 years and treated with lifestyle modification±metformin  G1 (Paleolithic diet alone - PD, n=15) G2 (Paleolithic diet with structured exercise - PDEX, n=13) | G1 (PD): - Frequency: five sessions (the first two meetings were held during the first two weeks after baseline measurements and thereafter once a month) - Setting: group - Staff: physician, trained dietician   G2 (PDEX): - Frequency: five sessions (the first two meetings were held during the first two weeks after baseline measurements and thereafter once a month) + three 1-h exercise sessions per week for 12 weeks (structured high intensity exercise intervention) - Setting: group - Staff: physician, trained dietician, educated personal trainer | Baseline and after 12 weeks | BMI change (kg/m2) G1: from 31.4 to 28.9 (P < 0.05) G2: from 31.4 to 29.1 (P < 0.05)  Both interventions were associated with a reduction in BMI (G1: −8%; G2: −7%; effect of time P<0.05) | Primary | Change from baseline  Waist circumference (cm) G1: 111 --> 100 (P < 0.05) G2: 107 --> 99 (P < 0.05) (G1: −10%; G2: −8%; effect of time P<0.05)  HbA1c (mmol/L) G1: 53 --> 42 (P < 0.01) G2: 56 --> 43 (P < 0.01) (G1: −21%; G2: −23%; effect of time P<0.01)  Fasting glucose (mmol/L) G1: 7.8 --> 6.3 (P < 0.01) G2: 9.2 --> 7.1 (P < 0.01) (G1: −19%; G2: −33%; effect of time P<0.01) |
| Watson et al.^32^ | Australia | 61 | 46% | Randomized trial | Adults (age range: 18–70 years, mean age = 55 years), BMI >= 25 kg/m2 (mean BMI = 34.35 kg/m2), 20% taking insulin, with type 2 diabetes  G1 (Higher-Protein Diet - HP, n = 32) G2 (Higher-Carbohydrate Diet - HC, n = 29) | G1 (HP): - Frequency: 12-week energy- restricted weight loss phase followed by a 12-week energy-balance weight maintenance phase. Dietetic appointments every two weeks + 30 min of moderate intensity aerobic exercise, 5 times per week (150 min/week). - Setting: individual - Staff: qualified dietitian  G2 (HC): - Frequency: 12-week energy- restricted weight loss phase followed by a 12-week energy-balance weight maintenance phase. Dietetic appointments every two weeks + 30 min of moderate intensity aerobic exercise, 5 times per week (150 min/week). - Setting: individual - Staff: qualified dietitian | Baseline, week 12 and week 24 | Weight (kg) change 0-12 weeks G1: -8.0 ± 0.80 G2: -7.6 ± 0.8  Weight (kg) change 12-24 weeks G1: -1.0 ± 0.6 G2: -0.2 ± 0.6  Weight (kg) overall change G1: -8.9 ± 1.3 G2: -7.7 ± 1.3  BMI (kg/m2) change 0-12 weeks G1: -2.80 ± 0.25 G2: -2.56 ± 0.26  BMI (kg/m2) change 12-24 weeks G1: -0.30 ± 0.19 G2: -0.01 ± 0.20  BMI (kg/m2) overall change G1: -3.10 ± 0.39 G2: -2.57 ± 0.40 | Secondary | HbA1c (%) change 0-12 weeks G1: -1.53 ± 0.20 G2: -1.30 ± 0.20  HbA1c (%) change 12-24 weeks G1: 0.19 ± 0.16 G2: -0.20 ± 0.16  HbA1c (%) overall change G1: -1.34 ± 0.21 G2: -1.50 ± 0.22  Fasting Glucose (mmol/L) change 0-12 weeks G1: -2.3 ± 0.5 G2: -2.8 ± 0.6  Fasting Glucose (mmol/L) change 12-24 weeks G1: 0.24 ± 0.3 G2: 0.30 ± 0.3  Fasting Glucose (mmol/L) overall change G1: -2.1 ± 0.6 G2: -2.5 ± 0.6  Fasting Insulin (mU/L) change 0-12 weeks G1: -8.1 ± 1.8 G2: -8.9 ± 1.9  Fasting Insulin (mU/L) change 12-24 weeks G1: -1.4 ± 1.2 G2: 0.9 ±1.3  Fasting Insulin (mU/L) overall change G1: -9.6 ± 2.1 G2: -7.9 ± 2.2  Waist circumference (cm) change 0-12 weeks G1: -8.5 ± 0.9 G2: -7.0 ± 0.9  Waist circumference (cm) change 12-24 weeks G1: -1.0 ± 0.6 G2: -0.6 ± 0.6  Waist circumference (cm) overall change G1: -9.6 ± 1.2 G2: -7.5 ± 1.2  SBP (mmHg) change 0-12 weeks G1: -7.9 ± 1.9 G2: -11.2 ± 2.0  SBP (mmHg) change 12-24 weeks G1: -4.4 ± 1.8 G2: 1.4 ± 1.8  SBP (mmHg) overall change G1: -12.3 ± 1.8 G2: -9.8 ± 1.9  DBP (mmHg) change 0-12 weeks G1: -6.2 ± 1.2 G2: -7.2 ± 1.2  DBP (mmHg) change 12-24 weeks G1: -1.5 ± 1.1 G2: 2.3 ± 1.1  DBP (mmHg) overall change G1: -7.7 ± 1.4 G2: -4.9 ± 1.4  Total cholesterol (mmol/L) change 0-12 weeks G1: -0.6 ± 0.1 G2: -0.2 ± 0.1  Total cholesterol (mmol/L) change 12-24 weeks G1: 0.3 ± 0.1  G2: 0.2 ± 0.1  Total cholesterol (mmol/L) overall change G1: -0.4 ± 0.1 G2: -0.03 ± 0.1  LDL-C (mmol/L) change 0-12 weeks G1: -0.3 ± 0.1 G2: -0.1 ± 0.1  LDL-C (mmol/L) change 12-24 weeks G1: 0.02 ± 0.1 G2: 0.1 ± 0.1  LDL-C (mmol/L) overall change G1: -0.3 ± 0.1 G2: -0.004 ± 0.1  HDL-C (mmol/L) change 0-12 weeks G1: -0.05 ± 0.03 G2: 0.02 ± 0.03  HDL-C (mmol/L) change 12-24 weeks G1: 0.1 ± 0.04 G2: 0.1 ± 0.04  HDL-C (mmol/L) overall change G1: 0.03 ± 0.03 G2: 0.1 ± 0.03  Triglycerides (mmol/L) change 0-12 weeks G1: -0.8 ± 0.2 G2: -0.5 ± 0.2  Triglycerides (mmol/L) change 12-24 weeks G1: 0.3 ± 0.2 G2: -0.03 ± 0.2  Triglycerides (mmol/L) overall change G1: -0.4 ± 0.2 G2: -0.6 ± 0.2 |
| Ziegler et al.^35^ | Germany | 26 | 46% | Randomized parallel-group pilot trial | Adults (age range: 18–69 years, mean age = 54 years), BMI >= 30 kg/m2 (mean BMI = 34.45 kg/m2), with type 2 diabetes  G1 (HF-RM+C, n = 13) G2 (LF+RM-C, n = 13) | G1 (HF-RM+C): - Frequency: weekly visits for 8 weeks - Setting: individual - Staff: medical staff  G2 (LF+RM-C): - Frequency: weekly visits for 8 weeks - Setting: individual - Staff: medical staff | Baseline and after 8 weeks | Weight (kg) change 0-8 weeks G1: -4.17 ± 0.86*** G2: -3.95 ± 0.75***  BMI (kg/m2) change 0-8 weeks G1: -1.36 ± 0.26*** G2: -1.31 ± 0.24***  ***P<=0.001 for the within-group change over time from baseline to 8 weeks | Secondary | Systolic BP (mmHg) change 0-8 weeks G1: -1.54 ± 4.50  G2: -5.33 ± 3.98  Diastolic BP (mmHg) change 0-8 weeks G1: 0.23 ± 3.96 G2: -5.08 ± 2.46  Total cholesterol (mmol/L) change 0-8 weeks G1: -0.35 ± 0.15 * G2: -0.20 ± 0.27  LDL cholesterol (mmol/L) change 0-8 weeks G1: -0.20 ± 0.13 G2: -0.05 ± 0.20  HDL cholesterol (mmol/L) change 0-8 weeks G1: -0.09 ± 0.05  G2: -0.04 ± 0.06  Fasting glucose (mmol/L) change 0-8 weeks G1: -0.25 (0.89) G2: -0.64 (1.66)**  Fasting insulin (pmol/L) change 0-8 weeks G1: -23.8 (59.1) G2: -13.7 (29.8)*  HbA1c (%) change 0-8 weeks G1: -0.60 (0.85)** G2: -0.50 (0.67)***  HbA1c (mmol/mol) change 0-8 weeks G1: -6.56 (9.3)** G2: -5.46 (7.37)***  * P < 0.05 for the within-group change over time from baseline to 8 weeks **P <=0.01 for the within-group change over time from baseline to 8 weeks ***P<=0.001 for the within-group change over time from baseline to 8 weeks |
